# Supramolecular Architectures Based on the Self-Assembly of Suberin Hydrolysate, Betulin, and Their Hybrids

**DOI:** 10.1021/acs.langmuir.5c01278

**Published:** 2025-07-15

**Authors:** Muhammad Farooq, Charlotte Zborowski, Paula A. Nousiainen, Jenni Tienaho, Risto Korpinen, Monika Österberg

**Affiliations:** † Department of Bioproducts and Biosystems, School of Chemical Engineering, 174277 Aalto University, Vuorimiehentie 1, 02150 Espoo, Finland; ‡ Production Systems, Natural Resources Institute Finland (Luke), Viikinkaari 9, FI-00790 Helsinki, Finland

## Abstract

Self-assembly offers a promising approach for producing functional nanomaterials from renewable biomass sources, as demonstrated in this study investigating two hardwood birch ( Roth) bark extractives: suberin hydrolysate (SH) and betulin fraction (BF). Using solvent inversion self-assembly with acetone, ethanol, and γ-valerolactone as solvents and water as an antisolvent, we prepared nanoparticles with tunable properties. Comprehensive characterization using FESEM image analysis revealed that SH formed predominantly rod-like structures (77–587 nm), while BF formed spherical particles (14–74 nm), with morphologies significantly influenced by solvent type and concentration. Coassembly of SH and BF (1:1) resulted in unique hybrid star-shaped nanoparticles, exhibiting both rod-like and spherical features. All nanoparticles demonstrated hydrophobic properties, with BF crystals achieving superhydrophobic surfaces (water contact angle 162° ± 8°) and BF NPs showing excellent water repellency (153° ± 2°) and maintaining water droplet shape without absorption for over 30 min. The nanoparticles showed significant antimicrobial efficacy against Gram-positive bacteria , with SH NPs demonstrating the highest inhibition. XRD analysis revealed that the self-assembly process enhanced crystallinity for both SH and BF, contributing to their improved functional properties. The ability to achieve such precise control over nanoparticle assembly of these heterogeneous, renewable biomass extractives represents a significant advancement in sustainable nanomaterial development, making them particularly suitable for functional coating applications.

## Graphical abstract

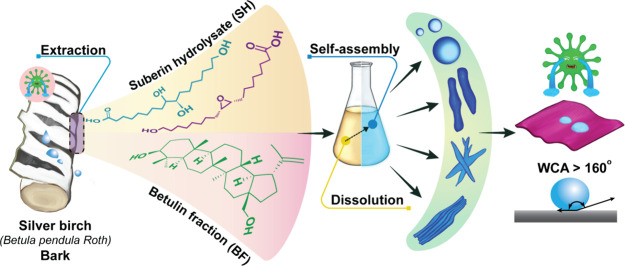

## 1 Introduction

From the formation of intricate cell walls in trees to the vibrant hues of hummingbird feathers, and even the complex structures of viruses and proteins, self-assembly is pervasive in biological systems.^1,2^ It allows individual components to organize into elaborate structures.^3^ Nature-inspired self-assembly using plant-based materials offers a route to sustainable solutions.^4,5^ The two major structural constituents of lignocellulosic biomass, namely, cellulose and lignin, have been extensively investigated for the development of nanomaterials.^4,6,7^ However, other wood extractives have received less attention due to their heterogeneity, structural complexity, and the need for additional purification, which makes their isolation and characterization challenging.^8^

Wood bark generates approximately 22 million tons annually as a side-stream in the European Union^9,10^ and is typically either landfilled or incinerated for energy generation.^11–13^ The outer bark of silver birch ( Roth) contains mainly suberin (35–44%) and triterpenoids (up to 40%).^14^ Suberin is rich in hydroxy and epoxy fatty acids, particularly 9,10-epoxy-18-hydroxy-stearic acid, while betulin is the predominant triterpenoid among other bioactive compounds like lupeol.^15,16^ Yet, few bark-derived products are commercially utilized.^11^

Suberin and its depolymerized hydrolysate (SH) have been proposed for various applications, including moisture barrier of cellulose substrates,^17,18^ hydrophobic coatings,^14,19^ adhesives,^20^ and as antimicrobial agents.^21,22^ Previous reports have either utilized dried bark extracts or their hydrolyzed monomers for antimicrobial applications^20,22,23^ or combined suberin fatty acid monomers with polymers and cross-linkers for biomedical and coating applications. However, the requirement for chemical cross-linkers and high processing temperatures (180–248 °C)^18,24^ emphasizes the need for more sustainable material design strategies.

Betulin and its derivatives exhibit diverse bioactivities, including antimicrobial and anticancer effects.^25^ Despite betulin’s bioactivity, research on betulin self-assembly remains scarce, with only a few notable studies investigating sonication-induced crystallization^26^ and betulin self-assembly in aromatic solvents.^27^ The practical utilization of betulin has been constrained by its low water solubility (0.08 μg/mL), high lipophilicity (log *P* value >9), and high molecular weight (>500 Da).^28,29^ Recent efforts to overcome betulin’s limited solubility include cyclodextrin complexation, which improved water solubility and bioavailability for hepatoprotective applications.^30^ Other approaches include nanoemulsion formulations^31,32^ and liposomal encapsulation,^33^ though these often require complex formulation processes or additional synthetic components. These challenges highlight the need for simpler, more sustainable approaches to prepare an aqueous dispersion of betulin while preserving its beneficial properties.

We hypothesize that the constraints associated with betulin and suberin extracts can be effectively addressed by incorporating supramolecular self-assembly principles. In this study, we leveraged our knowledge of lignin colloidal particle formation^34,35^ to control the morphology of birch bark extracts, specifically depolymerized suberin hydrolysate (SH) and betulin-rich (BF) fraction. Using solvent inversion self-assembly, we produced SH and BF nanoparticles in varied shapes and explored solvent effects on the self-assembly. SH and BF coassembled into star-like hybrid nanoparticles without requiring compatibilizers. Additionally, we examined BF crystallization in acetone and ethanol and used SH, BF, and hybrid particles to create superhydrophobic antibacterial coatings on cellulosic substrates. Our approach transforms these highly hydrophobic compounds into stable water dispersible nanoparticles and crystals without requiring chemical modifications, compatibilizers, or high-energy inputs. This creates environmentally friendly, water-based systems that retain the materials’ functional properties while enabling applications where aqueous compatibility is essential and organic solvents are undesirable.

## 2 Results and Discussion

In order to make conjectures regarding the self-assembly process and the morphology of architectures formed from SH and BF, it is necessary to consider their chemical structure and the potential interactions that may arise from the key functional groups. This approach provides insights into the driving forces behind the self-assembly process and aids in understanding the resulting structures.

### 2.1 Chemical Composition of SH and Betulin Fraction (BF)

First, the chemical compositions of the birch extracts were determined using chromatographic and spectroscopic analytical methods to correlate chemical composition with self-assembly behavior. The two birch bark fractions, obtained through a sequential ethanol–water extraction and an ethanolic alkaline hydrolysis, were analyzed using capillary GC–MS and GC-FID to identify their chemical composition and quantify the relative amounts of compounds (mg/g) present in the hydrolysates (Table S1). Additionally, both fractions were analyzed by 2D NMR for structural characterization (Figure 1) and by ^31^P NMR (Table 1) to quantify the aliphatic, aromatic, and carboxylic hydroxyl groups (mmol/g). These functional groups play a decisive role in both the self-assembly and the performance of the nanomaterial.

**Figure 1:**
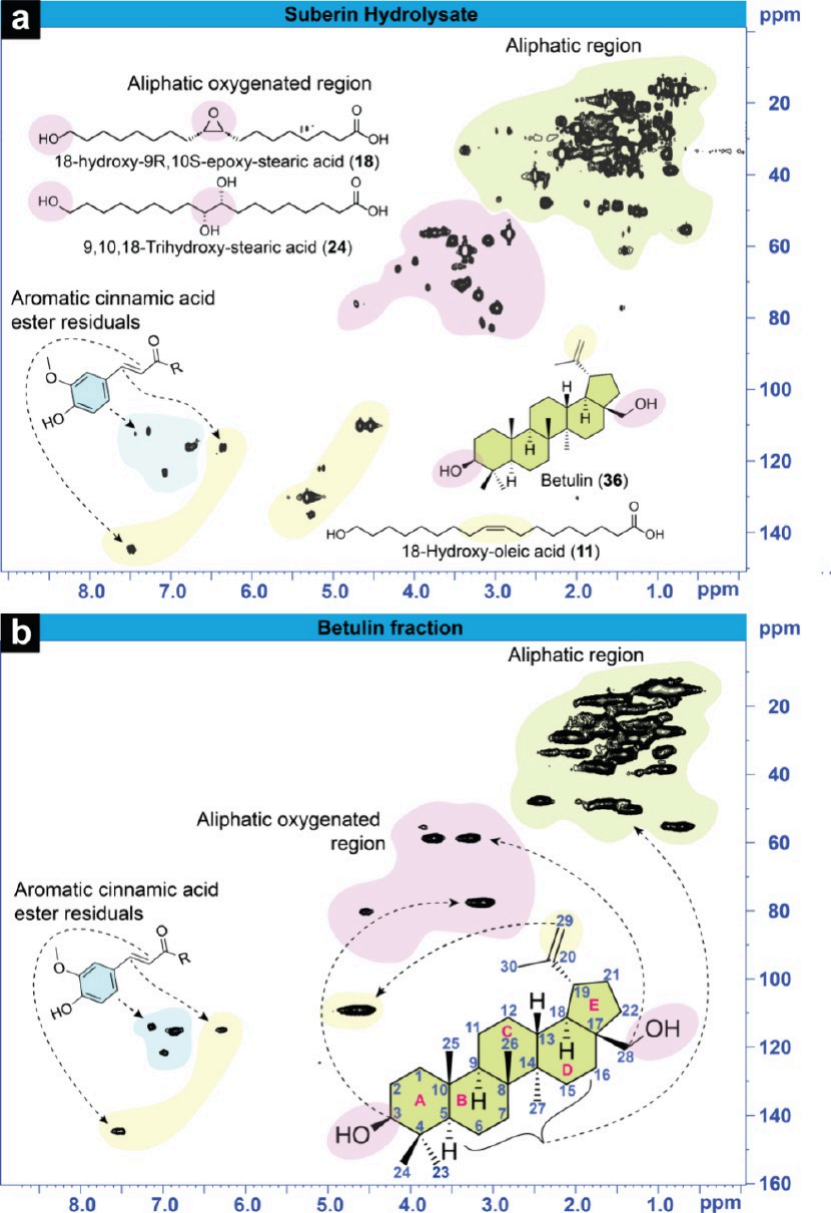
(a) HSQC NMR spectra of suberin-rich (SH in DMSO-*d*_6_) and (b) betulin-rich (BF in acetone-*d*_6_) fractions. The most prominent compounds in the fractions based on GC–MS analysis (Table S1) and their structural units are highlighted.

**Table 1:** Various Hydroxyl Group Contents of SH Hydrolysates and BF, as Determined by ^31^P NMR Analysis

functional group/measurement	BF (mmol/g)	SH fraction (mmol/g)
aliphatic-OH	1.36	1.36
condensed guaiacyl or 5-substituted-OH	0.00	0.02
guaiacyl-OH	0.16	0.16
p-OH phenyl	0.03	0.00
COOH	0.20	2.40
total phenolic	0.19	0.18
total OH	1.75	3.94
percentage of total hydroxyl content (%)		
phenolic-OH	11%	5%
aliphatic-OH	78%	34%
carboxylic acid	11%	61%

The chromatographic analyses demonstrated the efficiency of these extraction methods to produce a suberin fatty acid-rich (SH) and a betulin-rich (BF) fraction (Table S1). The GC–MS analysis of the SH fraction showed up to 77% volatilized low-molecular-weight compounds, of which 88% were identified. The identified compounds were various C16–C24 fatty acids (66%) and triterpenoids (34%). In SH, the most prominent hydrolyzed fatty acids were epoxy acids, such as 9,10-epoxy-18-hydroxy-stearic acid (**18**) in amounts of 160 mg/g, accounting for 23% (w/w) of the total identified compounds’ mass, along with a range of saturated and unsaturated C16–C24 hydroxy acids. These include tri-OH C18 acid (**24**), C22 acid (**25**), and unsaturated C18 acid (**11**), present in amounts of 74, 51, and 47 mg/g of the SH fraction. However, the largest constituent of the SH fraction was the pentacyclic triterpenoid betulin (**36**), accounting for 176 mg/g of the SH fraction. The BF fraction contained 98% volatile components, of which 99.4% was identified as triterpenoids and phytosterols. Interestingly, up to 64% of all of the identified compounds was betulin, together with other lupane-type compounds, with an extended propene group attached to the pentacyclic ring structure and olenane-type compounds with a double bond in the cyclic structures. Only 0.6% of the compounds identified in BF were classified as fatty acids.

The composition, especially in SH, was verified with 2D NMR spectroscopy. The HSQC NMR spectra of SH (Figure 1a) showed various strong correlation signals in the aliphatic region at δ_H_/δ_C_ 0–3/0–60 ppm. These signals originate from fatty acid and triterpenoid fused ring structures that could not be defined. In the middle region (at δ_H_/δ_C_ 3–5/50–90 ppm), where oxygenated CH correlation signals appear, the numerous signals indicate the variety of compounds in the mixture, including those with hydroxyl and epoxy groups. The aromatic region signals (at δ_H_/δ_C_ 6–8/100–130 ppm) and double-bond region (at δ_H_/δ_C_ 4–8/100–150 ppm) indicate the presence of a cinnamic acid aromatic structure, likely ferulic acid or its esters. Signals corresponding to double bonds of both betulin-type compounds at δ_H_/δ_C_ 4.6/110 ppm and unsaturated fatty acids at δ_H_/δ_C_ 5.3/130 ppm were visible in the SH sample. In BF, the betulin signals were highly prominent in both the double-bond and aliphatic regions, as well as the cinnamic acid-type aromatics as depicted in Figure 1b. The cinnamic acids were not detected in GC analysis, which suggests that they were bound in nonvolatile structures or larger molecules that were removed during sample preparation. No signals in the region for unsaturated fatty acids were observed in the BF sample, illustrating the high success of purification of this terpene fraction. The quantitative information on the amounts of hydroxyl groups in the fractions, obtained by phosphorus NMR, showed that the SH fraction contained a high number of carboxylic acids, up to 61% of the total OH groups (Table[Table tbl1]). On the other hand, BF mainly contained aliphatic OH groups accounting for 78% of the total hydroxyls and only a small amount of carboxylic acids that aligned also with the amount of aromatic phenols. Both fractions contained aromatic hydroxyls, most likely originating from cinnamic acids, as suggested by both GC–MS and HSQC NMR.

The main cinnamic acid present in SH and BF was guaiacyl-type ferulic acid, with possibly a minor amount of *p*-coumaric acid found in BF. However, in BF, the phenols constituted 11% of the overall hydroxyl groups, whereas in SH, the aromatics comprised only 5% of the total hydroxyl content. Since the hydroxyls represent polar groups in the molecules, their presence and amounts have a high impact both on molecule solubility in organic solvents and on further particle morphology during the self-assembly discussed in the next section.

### 2.2 Supramolecular Self-Assembly of SH

Given that the preparation of SH NPs via solvent inversion self-assembly has received only limited attention, we investigated this process and the effect of SH concentration (0.2–1 wt %) in different solvents on nanoparticle properties. Solvent selection plays a crucial role in self-assembly, influencing precipitation kinetics and morphology. Acetone, ethanol, and γ-valerolactone were selected based on their water miscibility, ability to solubilize the extracts, relatively low toxicity, accessibility for potential scale-up, and sustainability considerations, with γ-valerolactone being a biobased green solvent. Their different physicochemical properties, especially viscosity variations and diffusion rates, provided valuable insights into solvent-dependent self-assembly mechanisms.

Using acetone, ethanol, and γ-valerolactone as solvents, DLS measurements confirmed nanoparticle formation in acetone and ethanol with comparable hydrodynamic diameters (*D*_h_) across all concentrations (Figure 2a). The *D*_h_ of SH NPs showed different trends across the solvents. In acetone, *D*_h_ decreased with increasing concentration (from 243 to 150 nm, 0.2–1 wt %). In ethanol, *D*_h_ remained within the range of 189–238 nm for all of the concentrations. In γ-valerolactone, *D*_h_ increased significantly with increasing concentration (from 283 to 841 nm). The DLS analysis was complemented with image analysis of FESEM micrographs using Fiji ImageJ from dried particles deposited on silica wafers (Figure 2b). A significant variation between DLS-measured *D*_h_ and image analyzed diameter (*D*) was found for SH NPs from acetone and ethanol at 0.2 wt %. Image analysis revealed that the average particle diameters for particles derived from acetone and ethanol were 77 ± 4 and 77 ± 12 nm, respectively, whereas an increase in SH concentration led to an overall increase in the average diameter across all solvent systems, as opposed to DLS measurements. Statistical analysis of the image analyzed diameter (Figure 2b) confirmed significant concentration-dependent increases for all three solvents: acetone (*F*(4,10) = 130.13, *p* < 0.0001), ethanol (*F*(4,10) = 56.07, *p* < 0.0001), and GVL (*F*(4,10) = 26.55, *p* < 0.0001). The larger particle sizes observed with DLS are mainly due to three factors: increased light scattering by larger particles, sensitivity to agglomeration, and the *D*_h_ in suspension being larger than the dry particle diameter, with aggregation being the primary cause.^36^

**Figure 2:**
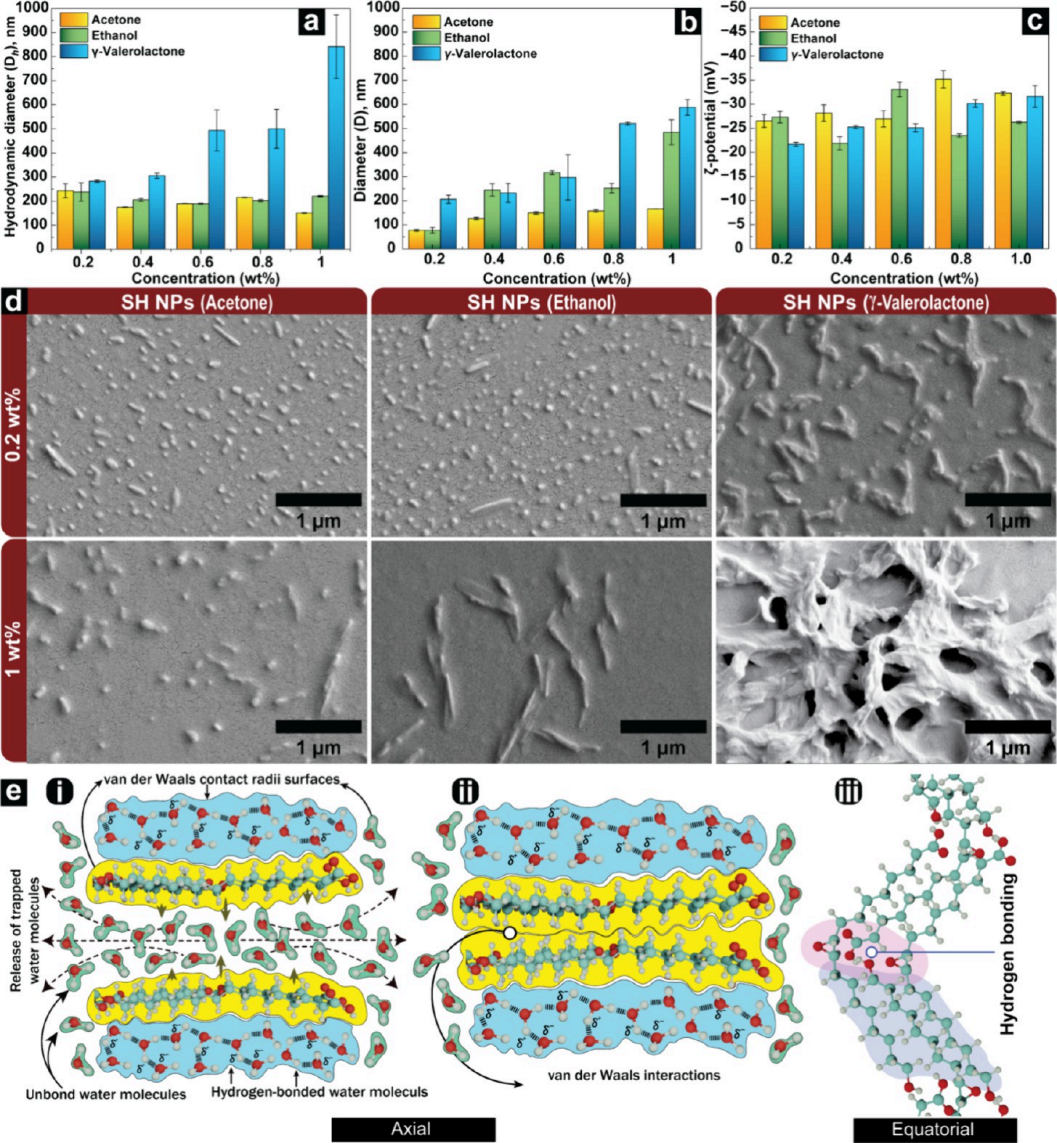
Characteristics of SH NPs obtained via self-assembly using acetone, ethanol, and γ-valerolactone as solvent systems and water as an antisolvent. (a) Hydrodynamic diameter (*D*_h_) values obtained from DLS measurements. (b) Diameter (*D*) values obtained from FESEM images using Fiji ImageJ. (c) Zeta potential values. (d) Representative FESEM micrographs of SH NPs produced at 0.2 and 1 wt %. (e) Proposed attractive interactions among extracted molecules (using 18-hydroxy-9R,10S-epoxy-stearic acid (**18**) as a model molecule).

Although all three solvents allowed SH NP preparation, significant differences can be observed between the sizes of nanoparticles. More specifically, the NPs produced from γ-valerolactone resulted in higher particle sizes compared with both acetone and ethanol. This difference in SH NP size can be attributed to the different diffusion rates of the solvent systems.^37^ In general, solvents with higher diffusion rates will allow faster transport of molecules to the aqueous phase, resulting in smaller NP sizes.^38^ The comparison of solvent viscosities reveals that acetone has the smallest viscosity value of 0.35 cP followed by ethanol 1.22 cP at 20 °C,^39^ whereas γ-valerolactone has the highest viscosity of 1.86 cP at 25 °C.^37^ The higher viscosity in the solvent phase has been postulated to translate into a lower diffusion rate of the solute molecules in the outer phase, resulting in an increase of SH NP particle size. Similarly, the effect of substrate concentration on particle size suggests a tendency of SH molecules to aggregate at higher concentrations, a phenomenon also observed during lignin self-assembly.^35^ The high viscosity and tendency of SH molecules to aggregate, even at low concentrations, are anticipated to impede the diffusion of the solvent at the interface. Hence, a smaller number of SH molecules will be transported into the aqueous phase, leading to the reduced formation of small SH particles at higher concentrations.^40^

Furthermore, an important factor influencing the size of nanoparticles in the solvent exchange process is the interactions between the solvent, antisolvent, and polymer. Zou et al.^41^ discovered through a molecular dynamics (MD) simulation study on lignin nanoparticles that a decrease in the interaction parameter between the solvent and the antisolvent, as well as an increase in the interaction parameter between the solvent and the polymer, leads to the formation of smaller nanoparticles.^41^ The self-assembly process induces the reorientation of functional groups at the particle surface, making surface charge characterization crucial for understanding colloidal stability. The zeta potential measurements revealed values ranging from −22 to −35 mV at pH 4–5 (Figure 2c, Table S2). SH structural analysis showed saturated and unsaturated C12–24 acids as primary components, indicating that the negative surface charge arises from terminal carboxylic groups.^42,43^ This negative charge leads to electrostatic double-layer repulsion, stabilizing the aqueous NP dispersion.

The FESEM micrographs presented in Figures 2d and S1 indicate that the shape of the nanoparticles is contingent upon both the concentration of SH and the solvent utilized. At a 0.2 wt % SH concentration, both spheroidal and rod-shaped nanoparticles coexisted in ethanol and acetone solvent systems. Increasing the SH concentration to 0.4 wt % in the ethanol solvent system resulted in the absence of spherical particles ( Figure S1). However, at 0.4 wt % SH concentration in the acetone-based system, spheroidal nanoparticles were still present to some degree. At SH concentrations ranging from 0.6 to 1 wt %, the predominant particle morphology observed in both solvent systems was a rod-like structure, although there were distinct variations in their morphology. Specifically, at an SH concentration of 0.6 wt % in ethanol, the formed nanoparticles exhibited a needle-like shape with a pointed end and a thicker middle, while the nanoparticles from acetone were cylindrical and also contained spheroids. The NPs, from both ethanol and acetone solvent systems, maintained their needle-like and rod-like shapes, even at higher concentrations of 0.8 and 1 wt %. In contrast, the nanoparticles obtained from the γ-valerolactone solvent system showed irregularly shaped bicontinuous structures with very few rod-like particles at lower concentrations, whereas at 1 wt %, they formed large network-like clusters instead of separate nanoparticles (Figure 2d). The NMR and GC–MS analyses (Tables 1and S1) confirmed the presence of several long-chain fatty acids. The major fatty acid constituent of the SH fraction was 18-hydroxy-9*R*,10*S*-epoxy-stearic acid (**18**), a derivative of a long-chain C18 fatty acid with multiple functional groups bonded to a hydrocarbon backbone, and is used in Figure 2e to illustrate possible attractive interactions during self-assembly. Self-assembly in aqueous systems involves cooperative intermolecular hydrogen bonding between water molecules, forming an intricate hydration network around solute molecules (Figure 2e­(i)). As the nonpolar groups of solute molecules come into contact, the water molecules preferentially interact with each other rather than with these groups. This restructuring of the hydration shell decreases water–solute contacts, altering the system’s free energy as a function of solute configuration and inducing indirect solute–solute interactions. The clustering of nonpolar moieties excludes intervening water (ii), bringing solutes into close proximity where van der Waals interactions play a key role in facilitating self-assembly (Figure 2e). While this phenomenon is commonly referred to as hydrophobic interactions, it is important to clarify that when hydrophobic moieties are first introduced into water, water molecules surrounding them adopt a more ordered structure, resulting in decreased entropy. Upon aggregation or self-assembly of these hydrophobic species, the ordered water molecules are released into the bulk phase, leading to an increase in the system’s entropy. This entropy gain is a major contributor to the favorable free energy change associated with hydrophobic aggregation.^44^

In the self-assembly of compound (**18**), as depicted in Figure 2e­(i,ii,iii), both hydrogen bonding and van der Waals forces are expected to play key roles. Functional groups like hydroxyl, carboxyl, and epoxy can form hydrogen bonds,^45^ although the aqueous environment might weaken these interactions.^46^ The other hydroxy fatty acids in the SH fraction, such as compounds (**24**), (**25**), and (**11**), are expected to assemble similarly. However, the SH fraction’s heterogeneity, composed of various fatty acids and pentacyclic terpenoids, significantly affects the self-assembly process. Prior studies on fatty acids and lipids have suggested that impurities can disrupt self-assembly.^47^ Functional group proximity and arrangement, such as closely spaced carboxylic groups, may further hinder assembly by causing steric hindrance or promoting intramolecular over intermolecular hydrogen bonding.^48^ Additionally, when the molecules with aromatic rings come within 5 Å of each other, attractive π–π stacking interactions between them can occur, further driving the self-assembly process. Together, these noncovalent forces direct the spontaneous assembly in aqueous environments.

Despite the inherent chemical heterogeneity, the self-assembly of SH in water still resulted in distinguishable particle morphologies, as illustrated in Figure 2d. It implies that while being a mixture of a variety of suberin fatty acids and triterpenoids rather than a single component system, SH has the potential to generate supramolecular structures with a tunable size and shape through the manipulation of solvent type and concentration. Now that we have established a foundational understanding of the chemical composition and self-assembly behavior of the SH fraction, we investigated the second most abundant component in birch bark extract by using the isolated BF consisting almost purely of triterpenoids. With an understanding of the factors that control SH self-assembly, we can use similar methods to uncover the formation processes of betulin nanostructures.

### 2.3 Supramolecular Self-Assembly of BF

Alongside the SH fraction, the BF was also extracted, yielding 36% of the original outer bark. We now turn to investigating the self-assembly behavior of the BF using the protocol previously established for SH NPs. The influence of different solvents (acetone, ethanol, and γ-valerolactone) and concentrations of BF on the particle size and morphology was systematically studied to achieve control over particle size and surface characteristics. The FESEM images presented in Figure 3a illustrate that the BF nanoparticles exhibit a uniform size distribution and consistent morphology across all tested solvents. The morphology of BF NPs can be best described as irregular spheroids. The size data extracted and analyzed using ImageJ software, presented in Figure 3b, reveal a marked difference in the size evolution and morphology between BF and SH nanoparticles. At 0.2 wt % concentration, BF self-assembly produced nanoparticles with diameters under 20 nm in all three solvents. However, further investigations at higher concentrations were focused on using acetone and ethanol as solvents due to γ-valerolactone’s limitations. At 0.4 wt % BF, particle sizes averaged 74 ± 1 nm in acetone and 49 ± 7 nm in ethanol. Statistical analysis of BF particle size data showed significant concentration-dependent effects for acetone (*F*(4,6) = 14.81, *p* = 0.003) but not for ethanol (*F*(4,6) = 4.14, *p* = 0.060). No change in average particle size was observed when increasing the BF concentration from 0.4 wt % to 1 wt %. However, particle morphologies became increasingly spherical, with complete spherical shapes achieved at 0.4 wt % in ethanol and 0.6 wt % in acetone, showing a solvent-dependent geometry (Figure S2). The self-assembled BF NPs exhibited a negative zeta potential (Figure 3c), with values of −32.3 ± 2 mV, −28.4 ± 1.2 mV, and −24.6 ± 0.8 mV for 0.2 wt % particles from ethanol, acetone, and γ-valerolactone, respectively. The pH of dispersions varied between 5.5 and 6.7, except for γ-valerolactone samples, which had a more acidic pH of 4.1 (Table S2). Although pure betulin lacks charged groups, the observed negative zeta potential can be attributed to betulinic acid and other charged extractives, which together constituted 11% of the total hydroxyl content of BF (Figure 1b,Table S1).

**Figure 3:**
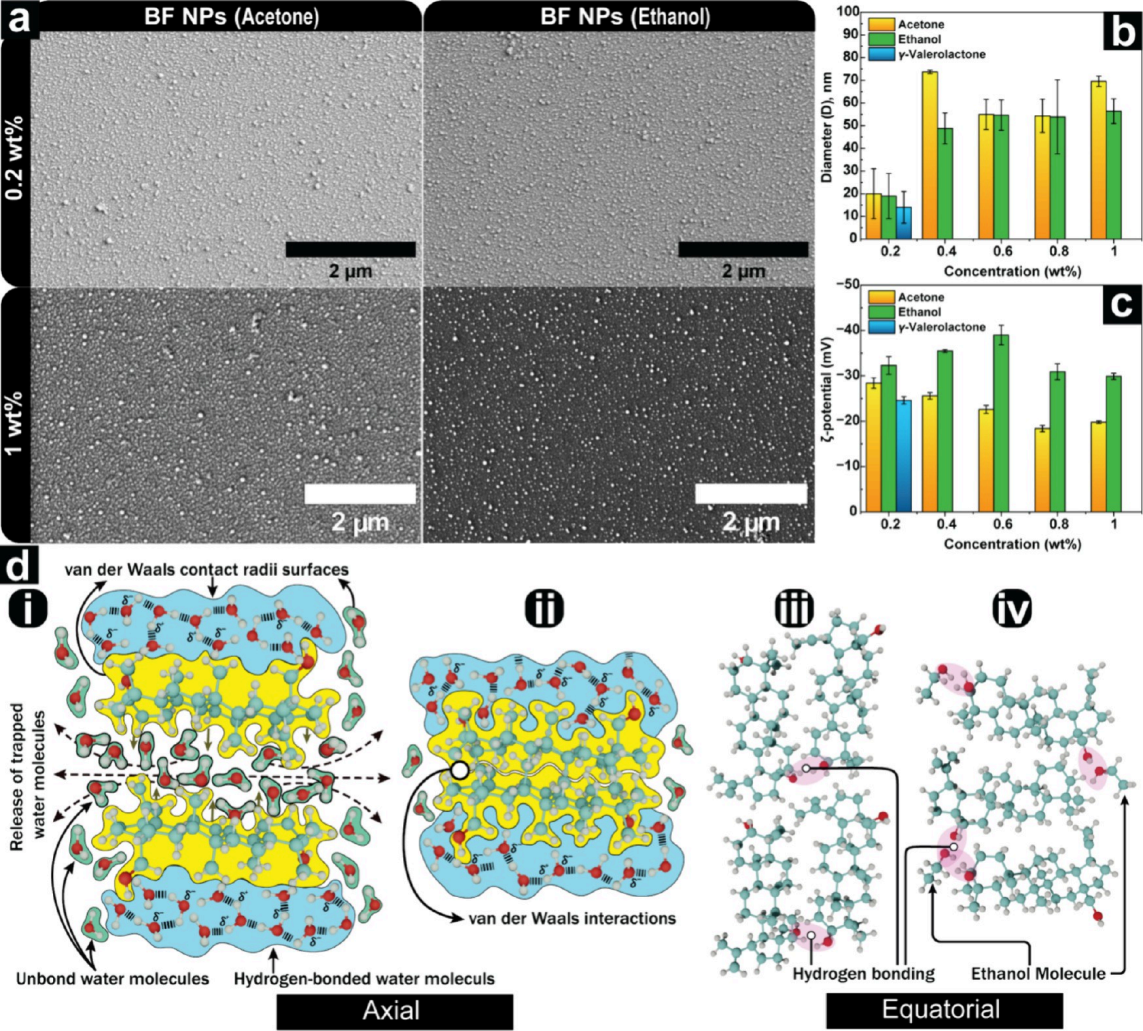
Characteristics of BF NPs obtained via self-assembly using acetone, ethanol, and γ-valerolactone as solvent systems. (a) FESEM micrographs of BF NPs at 0.2 wt %. The scale bar is 2 μm for the magnified micrographs. (b) Diameter values obtained from FESEM image analysis using Fiji ImageJ. (c) Zeta potential values. (d) Proposed hypothetical illustrations (i–iv) of the self-assembly mechanism of betulin.

The self-assembly of the betulin-rich fraction (BF) into nanoparticles results from noncovalent interactions among the extract components, water, and organic solvent. Due to system complexity, we can only hypothesize the main driving forces. Betulin (**36**) is a lipophilic compound with a rigid, nanosized structure measuring 1.29 nm in length,^27^ characterized by a hydrophobic triterpene backbone composed of fused pentacyclic rings.^49^ The pentacyclic rings exhibit a nonpolar character, which facilitate their aggregation in nonpolar solvents.^27^ The hydrophilic characteristics of betulin are ascribed to the existence of hydroxyl groups located at the “A” ring and a hydroxymethyl group at the ring junction of the trans-fused “D” and “E” rings (Figure 1b).^50^

During the self-assembly of betulin, the presence of water in the system promotes interactions between the hydroxyl groups and water molecules. Meanwhile, water molecules in the vicinity of betulin molecules rearrange and move toward the bulk phase, which brings the fused pentacyclic structures of betulin closer together. This leads to the formation of a rigid, planar stack, facilitating van der Waals interactions (Figure 3d­(i,ii)).^27^ The OH groups that are arranged parallel or nearly parallel are involved in hydrogen bonding, thus complementing the formation of a hydrophobic outer core (Figure 3d­(iii)).^27^ The compositional analysis of natural extracts such as BF and the structural influence on the physicochemical properties that facilitates supramolecular self-assembly are significant factors in determining the nanoparticles’ distinctive properties and potential applications in various fields where stable dispersion in physiological conditions is required.

### 2.4 BF Crystals

The crystallization of the betulin-rich fraction at higher concentrations allowing for supersaturation and nucleation is of interest due to its potential for forming structured materials. The FESEM micrographs presented in Figure 4a,b illustrate that the crystallization process of BF initiates at significantly lower concentrations when using acetone compared to ethanol. This is further supported by the accompanying photographs of the corresponding suspensions presented in Figure 4c,d. The length of the BF crystals is measured from FESEM micrographs using Fiji ImageJ software. From acetone solutions, the formation of BF crystals was observed at a concentration as low as 2 wt %. These crystals had an average length of 1 ± 0.5 μm and a thickness of 60 ± 24 nm. In addition to the crystals, small spherical particles (∼40 ± 20 nm) were formed through the self-assembly of the BF solution upon exposure to water during the process of crystal transfer for visualization. A linear trend was observed in crystal length as the concentration increased, with crystal sizes growing progressively from 3 to 5 wt %. The thickness remained within the range of 49 ± 13 nm to 66 ± 18 nm, except at the highest concentration of 6 wt %, where a noticeable increase in thickness was observed, and crystal bundles formed instead of individual crystals (Table S3). Unlike in acetone, crystallization was not observed in ethanol at 2 and 3 wt % concentrations. Instead, only spherical particles were present, measuring 55 ± 35 and 54 ± 3 nm in diameter, indicating a leveling-off point for particle growth. Upon examination of the micrograph of BF at 4 wt % concentration, a limited number of small crystallites appeared, with an average length of 200 ± 75 nm, and at 5 wt %, a notable enhancement in crystal size with an average length of 662 ± 276 nm was obtained.

**Figure 4:**
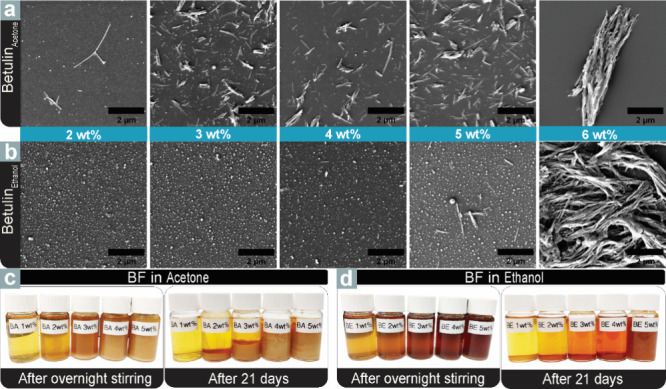
Self-assembled BF precipitates from acetone and ethanol dispersed in water after 21 days for FESEM visualization. FESEM micrographs of BF precipitates in (a) acetone and (b) ethanol. The scale bar is 2 μm. (b) Photographs of BF dissolved in (c) acetone and (d) ethanol after overnight stirring and after 21 days.

However, the BF crystals formed in the presence of ethanol at a 6 wt % concentration exhibited an atypical morphology when compared to their formation in the presence of acetone. Specifically, the ethanol system exhibited a fibrillar network structure, while the crystals from acetone were more rigid and exhibited a distinct orientation in their shape. The mean length of a crystal bundle obtained from acetone is estimated as 5.9 ± 1.5 μm, whereas the fibrillar crystal structure of the ethanolic system is approximately over 10 μm in length. Betulin exhibits higher inherent solubility in acetone compared to ethanol at 298.2 K.^51,52^ However, an increased formation of betulin crystals was observed in acetone solutions, as opposed to those prepared with ethanol. While acetone provides superior equilibrium solubility, its weaker interactions with betulin molecules facilitate molecular mobility and reduce nucleation barriers, promoting crystallization.^53^ This kinetic dominance is further complicated by the presence of impurities in the semipurified BF, notably monogynol A (**42**), betulinic acid (**37**), and lupeol (**30**), which can act as heterogeneous nucleation sites or crystal growth modifiers, effectively overriding the thermodynamic solubility considerations. Conversely, ethanol’s robust hydrogen bonding network creates stable solvation shells that inhibit the molecular reorganization necessary for crystal formation, suggesting that the crystallization was governed by kinetic factors rather than merely thermodynamic solubility.

The dissimilarity in the crystal morphology obtained from acetone and ethanol can be attributed to the distinctive chemical properties of the solvent molecules. Acetone, a polar aprotic solvent, acts as a proton acceptor. In the crystals derived from acetone, the molecule accepts a hydrogen bond from the neighboring hydroxyl group of the betulin molecule, resulting in the formation of an infinite one-dimensional hydrogen bonding chain. Ethanol, in contrast, is classified as a polar protic solvent that exhibits the ability to act as both a proton acceptor and a proton donor. During the crystallization event, the ethanol molecules formed two-dimensional hydrogen bonding networks with betulin. The OH groups of ethanol readily donate and accept hydrogen bonds with the OH groups of betulin, thereby creating a bridge between the neighboring betulin molecules. Through the utilization of complementary solvent-betulin hydrogen bonds, the ethanol successfully facilitated the connection of betulin molecules, resulting in the formation of continuous sheets,^26,54^ as depicted in Figure 3d­(iv).

### 2.5 SH–BF Hybrid Nanoparticles (SB NPs)

The supramolecular self-assembly of SH and BF was a result of their unique chemical structures. This observation enticed us to develop a system that combines spherical and needle-like morphologies in order to leverage the advantages of both morphologies. First, the hybrid nanoparticle systems were constructed by using the 0.2 wt % concentration of BF and SH and subsequently mixing them at varying ratios, followed by self-assembly in water as antisolvent (Figure 5a). Next, we fixed the SH to BF ratio to 1:1 and varied the concentration of each solution from 0.2 to 1 wt % (Figure 5b) in order to investigate the impact of concentration on the morphology of formed constructs. A distinct pattern as a function of the SH:BF ratio is evident from FESEM micrographs presented in Figure 5a. At 1:9 and 3:7 ratios of SH to BF, the hybrid nanoparticles maintained BF’s characteristic spherical morphology, with similar sizes of 25 ± 13 nm and 25 ± 12 nm, respectively.

**Figure 5:**
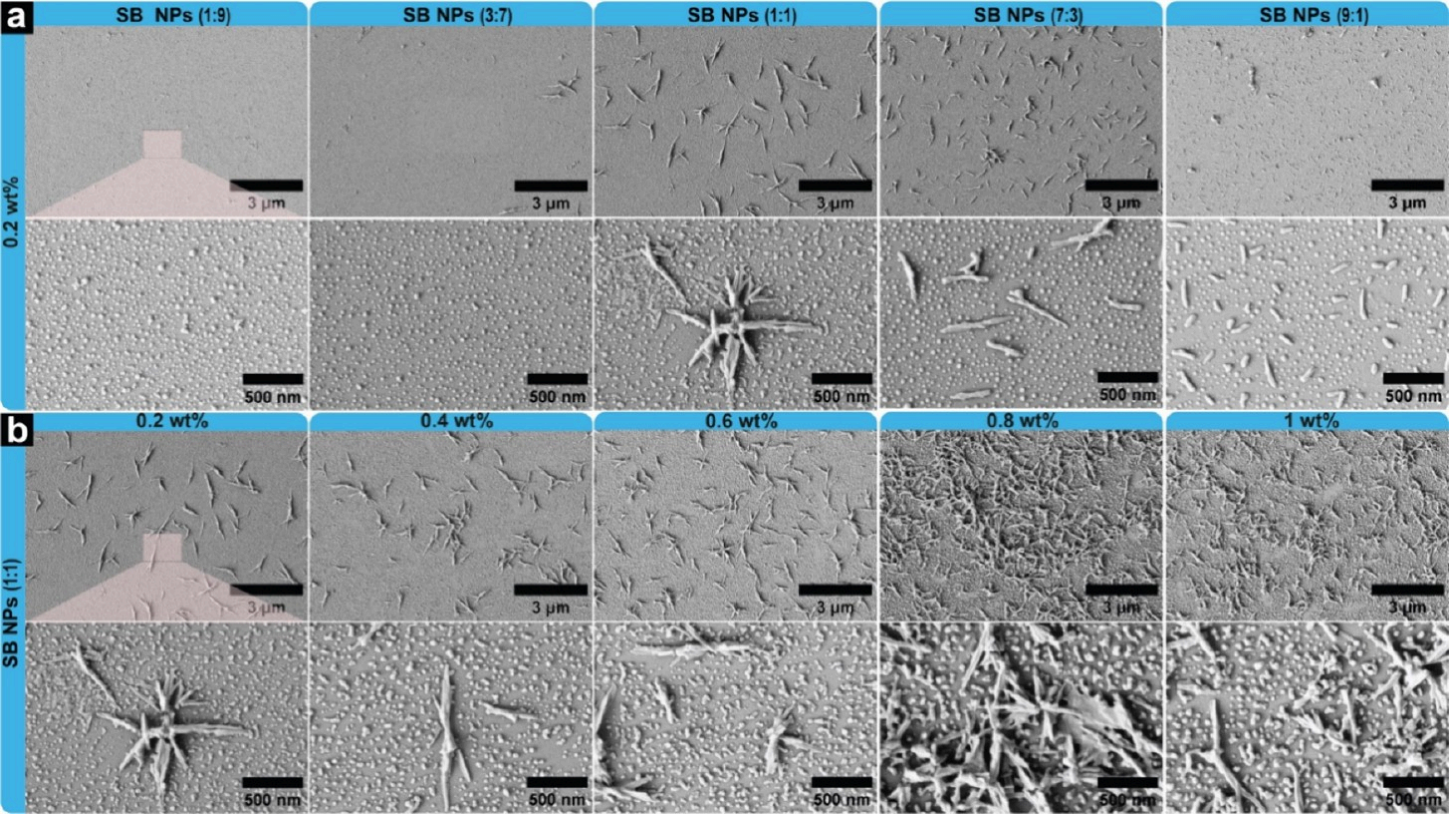
Self-assembled hybrid SB NPs from acetone. FESEM micrographs of hybrid SB NPs (a) at different SH to BF ratios at a fixed 0.2 wt % concentration and (b) at a fixed SH to BF ratio of 1:1 at different concentrations. The scale bar is 3 μm for the full-field micrographs and 500 nm for the magnified micrographs.

At 1:1 ratio, star-like structures emerged with needle-shaped features (870 ± 270 nm long), while spherical particles transformed into clusters averaging 39 ± 10 nm in diameter. These star-shaped structures displayed a fibrillar morphology radiating from a central point with sharp-ended needles. At 7:3 SH:BF ratio, stars transformed into singular needles (559 ± 191 nm) and clusters reverted to dome-like spheres (23 ± 12 nm). Further increasing SH content to 9:1 resulted in shorter, cylindrical structures (135 ± 49 nm) without sharp edges, coexisting with dome-like particles (30 ± 13 nm).

Since the 1:1 ratio of SH and BF resulted in two morphologies with unique characteristics, we further examined the effect of concentration on their structural features. Figure 5b shows micrographs of hybrid nanoparticles from 0.2 to 1 wt % concentrations at this fixed ratio. At concentrations below 0.6 wt %, needle-like and spheroid morphologies coexist, with nearly identical characteristics. At 0.8 and 1 wt %, the star-shaped morphology becomes more prominent, forming a network. The average length of needle structures below 0.6 wt % was 859 ± 7 nm, with spheroids around 41 ± 7 nm. At 0.8 and 1 wt %, the spheroids measure 23 ± 12 and 30 ± 13 nm, but needle lengths were unmeasurable due to network formation. These systematic morphological transitions reveal how the interplay between SH and BF during self-assembly can be precisely tuned by controlling the composition and concentration. The resulting hierarchical structures, particularly the star-shaped morphologies and their networks, could be valuable for applications requiring enhanced surface roughness such as superhydrophobic coatings and high-surface-area catalysts.

### 2.6 Structural Properties of SH, BF, and Their Self-Assembled Architectures

The process of supramolecular self-assembly involves the organization of molecules into well-defined structures through noncovalent interactions, which can greatly influence the resulting crystalline or amorphous nature of a material. Thus, we examined the impact of the self-assembly processes of SH and BF on the molecular conformation in the resultant morphologies. The multiple maxima in the XRD spectra of SH powder indicate a semicrystalline structure with distinct reflections at around 7° and 9° (2θ), along with several reflections within the 14°–31° (2θ) range, Figure 6a. These reflections are specifically observed at 14°, 20°, 21°, 23°, 24°, 26°, 30°, and 31° (2θ).

**Figure 6:**
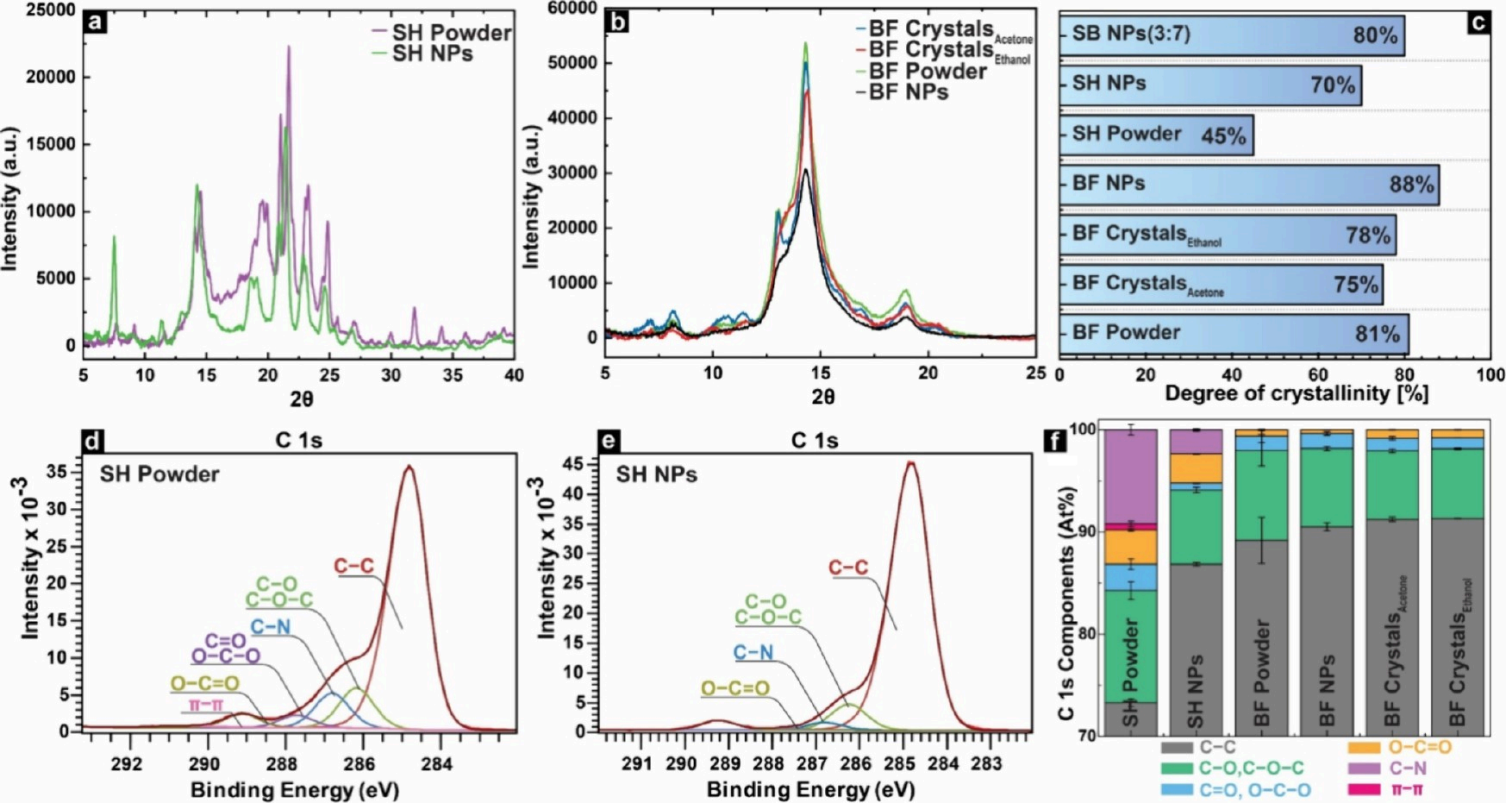
Structural characteristics of SH, BF, and their various forms. XRD diffractogram of (a) SH starting powder and NPs, (b) BF starting powder, crystals, and NPs, and (c) Degree of crystallinity. (d) XPS C 1s spectra of SH and (e) SH NPs. (f) Atomic percentages of C 1s components of SH, BF, and their self-assembled counterparts.

The XRD analysis of SH powder shows small crystalline domains alongside amorphous regions, consistent with XRD patterns of long-chain C10–C18 alcohols, epoxides, and acids reported by Sousa et al.^55^ and Heinämäki et al.^16^ GC–MS analysis identified epoxy C18 acid (**18**) and trihydroxy C18 acid (**24**) as the primary constituents of SH, supporting these XRD findings. The SH powder exhibited 45% crystallinity (Figure 6c), which increased to a higher degree of ordering (70%) when self-assembled into nanoparticles, as indicated by sharper and narrower XRD peaks. This enhanced ordering likely resulted from the purification process, dissolving and centrifuging SH to remove undissolved material. The removal of impurities was confirmed by XPS results showing a reduction in nitrogen-containing structures from 10% in the powder to significantly lower levels in the self-assembled form (Figure 6d–f).

Betulin displays multiple pseudopolymorphic forms,^56^ highlighting the importance of analyzing the crystal structure of the diverse morphologies produced in this study. The initial powder of the BF as well as the self-assembled nanoparticles and crystals of the BF obtained from ethanol and acetone were subjected to XRD analysis. The diffractograms obtained for all the examined structures of BF presented in Figure 6b demonstrate similar patterns in the 5°–25° (2θ) range, characterized by two distinct reflections at approximately 19° and 14° (2θ).^56,57^ BF powder and betulin crystals derived from acetone showed an additional peak at 13°, which was absent in ethanol-derived crystals and BF NPs. Instead, these showed a peak shoulder at the same angle, with BF NPs displaying reduced shoulder dimensions. The recrystallization of betulin from different solvents has been reported to lead to the formation of distinct crystalline structures. For instance, when ethanol-betulin solvate is dried at room temperature, it can lead to the formation of betulin hemihydrate.^58^ Therefore, it is reasonable to attribute these variations in diffractograms observed in betulin crystals obtained from acetone and ethanol to betulin polymorphism.

The calculated degree of crystallinity for all the produced BF specimens falls within the range of 75% to 88% (Figure 6c). The initial BF powder displayed a degree of crystallinity of 81%, while the BF crystals obtained from acetone and ethanol showed slightly lower degrees of crystallinity of 75% and 78%, respectively. The degree of crystallinity values determined for the BF NPs is notably high, 88%, surpassing that of other betulin-derived structures. This finding is significant because it suggests that the nanoparticles have the potential for enhanced stability and efficacy in various applications. Higher purity starting material leads to increased crystallinity, as seen in BF NPs, where the removal of undissolved fragments during the synthesis enhances crystallinity. The XRD pattern of BF NPs exhibits a lower intensity in comparison to that of the BF powder or crystals, even though the degree of crystallinity is higher. Such observation was earlier reported by Zhao et al.^57^ This can be due to the fact that smaller and more numerous crystallites exist in BF NPs due to the nanoscale dimension of particles, which results in overall lower peak intensity. Second, if a significant proportion of the crystallites within a material exhibit an orientation that impedes efficient X-ray diffraction, this can result in reduced peak intensities, despite the presence of numerous well-organized crystallites.^59^

Followed by XRD, the surface chemistry of the SH and BF structures was investigated using XPS. Due to its surface sensitivity of approximately 3 nm for organic compounds, XPS can provide vital information about the chemical makeup of the surfaces resulting from the self-assembly procedure.^60^ The C 1s spectra of both the SH powder and the self-assembled SH NPs are depicted in Figure 6d,e, respectively. Additionally, relative percentages of the C 1s components are presented in Figure 6f. As anticipated, the measured surfaces were found to be predominantly composed of carbon, with the most prominent peak (C 1s) observed at 284.8 eV (Figure 6d,e). This peak corresponds to the presence of sp^3^- and sp^2^-hybridized aliphatic/aromatic carbon that is bonded solely to carbon or hydrogen.^61^ Furthermore, the spectrum displays distinct peaks corresponding to carbon atoms that are single-bonded to oxygen (C–O at 286.3 ± 0.3 eV), carbon atoms double-bonded to oxygen (O–C–O, C=O at 287.7 ± 0.1 eV), and carbon atoms both single- and double-bonded to oxygen (O=C–O at 289.1 ± 0.2 eV).^62^ The process of supramolecular self-assembly of SH into nanoparticle SH NPs leads to an augmentation in the C–C component. On the other hand, the O–C–O, C=O, and O–C=O components exhibited a decrease. The SH also exhibited a π–π* shakeup satellite peak at 290.6 ± 0.1 eV, indicating the presence of aromatic or other conjugated unsaturated carbon systems, such as the ferulic acid residues verified by GCMS and NMR analyses.

SH powder contained a significant C–N component that was not detected by the other techniques. Most likely, these originated from lipid-bound proteins or nitrogen compounds and were partially removed during processing since the SH NPs exhibited a significant reduction in nitrogen-component, accompanied also by reduction of π–π* contribution. During NP preparation, solvent dissolution and subsequent centrifugation separate soluble suberin from the insoluble residues that are removed prior to synthesis of NPs. This process concentrates long-chain fatty acids and aliphatic components in the NP core while excluding small hydrophilic constituents. On the other hand, the analysis of the relative percentages of components in the BF and BF NPs reveals negligible differences (Table S4), while the BF crystals obtained from acetone and ethanol exhibit a slight increase in the C–C component and a slight decrease in the C–O and C–O–C components. It is likely that the impurities trapped inside the crystal lattice during crystallization result in betulin’s pentacyclic structure dominating the surface, leading to a higher C–C signal. XPS survey scan and C 1S spectra of SH, BF, and their different morphologies are depicted in Figure S3.

### 2.7 Wetting Properties of SH, BF, and Their Self-Assembled Architectures

Inspired by their role as protective barriers within the plant cell wall, we studied the wetting characteristics of the various SH and BF architectures developed herein. The dispersions were attached to textile fabrics using a layer of cationic starch as a cationic polyelectrolyte to increase electrostatic interaction between the cellulosic fabric surface and NPs, and subsequently, their wetting properties were assessed.

The BF crystals exhibited remarkable wetting properties when coated on Tencel fabric, as evidenced by a contact angle (WCA) value of 162 ± 8° after 60 s of contact time (Figure 7a,c). The wetting behavior of BF crystal surfaces can be characterized as superhydrophobic when the water contact angle is 150° or greater.^63^ The superior hydrophobicity can be attributed to the surface chemistry and unique needle-like structure of the betulin crystals. The spiky morphology introduces surface roughness, leading to a Cassie–Baxter state that traps air and results in the exceptionally high WCA observed for BF crystals.^64,65^

**Figure 7:**
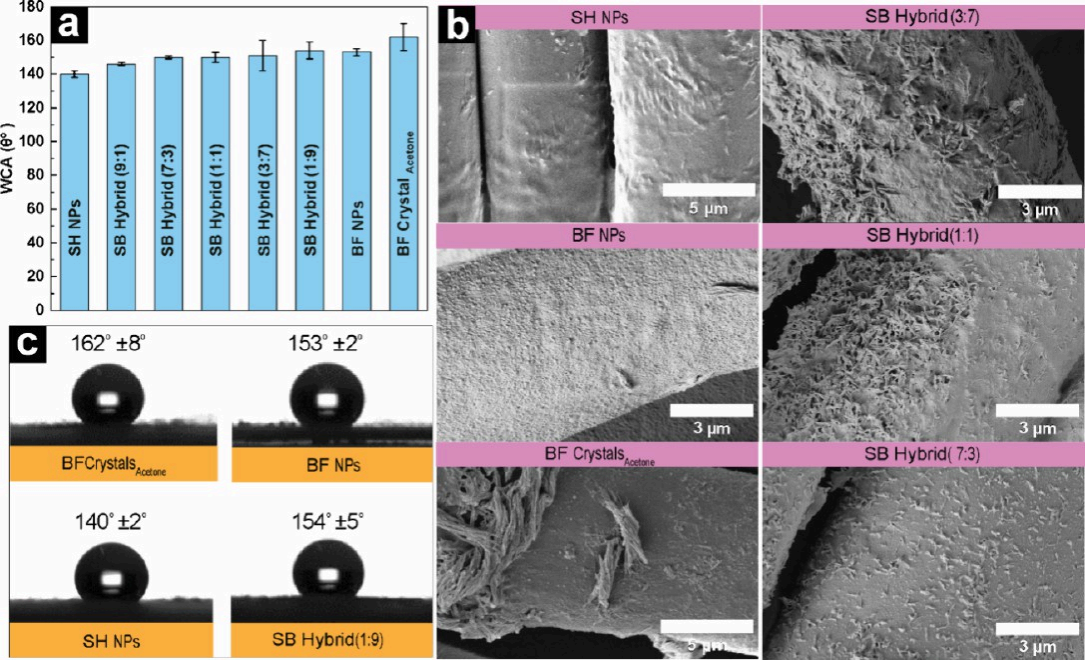
Wetting properties of SH NPs, BF NPs, BF crystal, and hybrid NPs. (a) Static WCA values plot measured for 1 min. (b) FESEM micrographs of SH NPs, BF NPs, BF crystals_Acetone_, and SB hybrid NPs. (c) Photographs of water droplet on TENCEL fabric during WCA measurement along with the respective WCA values measured for 1 min.

The XRD analysis of BF crystals revealed a significant presence of C–C components, correlating with the observed values of the WCA (Figure 7a). It is noteworthy that the BF fraction comprises only 64% betulin and overall, 95% triterpenoids of all identified compounds. The BF crystal production process is efficient, involving simple mixing and redispersion in water after crystallization. This straightforward approach to produce crystals does not require temperature control or high purity and justifies its facile processability while imparting excellent hydrophobic properties. Additionally, the self-assembled BF nanoparticles exhibit a highly competitive WCA value of 153 ± 2°. The results indicate that the hydrophobic nature of the nanoparticles was not compromised during the process of supramolecular self-assembly, despite the molecular reconfiguration. The surface of BF NPs contains a significant amount of hydrophobic functional groups, resulting in exceptional water repellency. The FESEM image of coated fibers in Figure 7b demonstrates that the nanoscale particle size and spherical geometry result in exceptional surface coverage of the substrate which is pivotal to obtain good hydrophobic surfaces. The SH NPs also exhibited a comparable hydrophobic character with a WCA value of 140 ± 2°, albeit slightly lower than that of BF crystals and NPs. Despite the notable hydrophobic nature, the FESEM image (Figure 7b) reveals that the SH NPs form a layer on the surface of the substrate fiber, as opposed to being dispersed as individual particles. The maintenance of the individual geometry of SH NPs was expected to result in an increase in surface roughness, thereby enhancing hydrophobicity and WCA values.^64^ With hybrid SB NPs, the WCA values remained similar across the varying betulin contents at SH to BF ratios of 7:3, 1:1, and 3:7 (Figure 7a). One-way ANOVA confirmed significant differences in WCA between sample types (*F*(7,16) = 4.81, *p* = 0.0045), with posthoc analysis identifying significant differences between BF crystals and both SH NPs (*p* = 0.001) and SB Hybrid 9:1 (*p* = 0.022). It is intriguing to note how two distinct morphological identities within a singular system synergistically contributed to the development of hydrophobic properties. For instance, at a SH to BF ratio of 7:3, the needle-like SH NPs and spherical BF NPs were all well-dispersed on the surface, contributing to the overall hydrophobicity of the surface. The reference TENCEL surface, coated with cationic starch, exhibited a superhydrophilic behavior with WCA less than 10°. This suggests that the deposition of BF and SH structures onto the TENCEL substrate can effectively tailor its surface properties in an economic and environmentally friendly way.

To evaluate the effectiveness of SH and BF NPs along with their hybrid NPs as water-repellent coatings on Tencel fabric, the WCA measurements were supplemented with observations of water droplet behavior over time (Figure 8). A water droplet was placed on the fabric surface, and its absorption was observed after 2, 15, and 30 min. Remarkably, BF NPs exhibited exceptional water resistance, maintaining a water droplet shape without absorption into the coated surface for 30 min. On the other hand, SH NPs and SB hybrid NPs at a 9:1 ratio displayed slight water absorption within 15 min, although droplets persisted on all coated surfaces for the first 15 min. After 30 min, complete absorption occurred on surfaces coated with SH NPs and SB hybrid NPs at 9:1, whereas fabrics coated with SB hybrid NPs at 7:3 and 1:1 showed slight absorption around the droplet perimeter (Figure 8).

**Figure 8:**
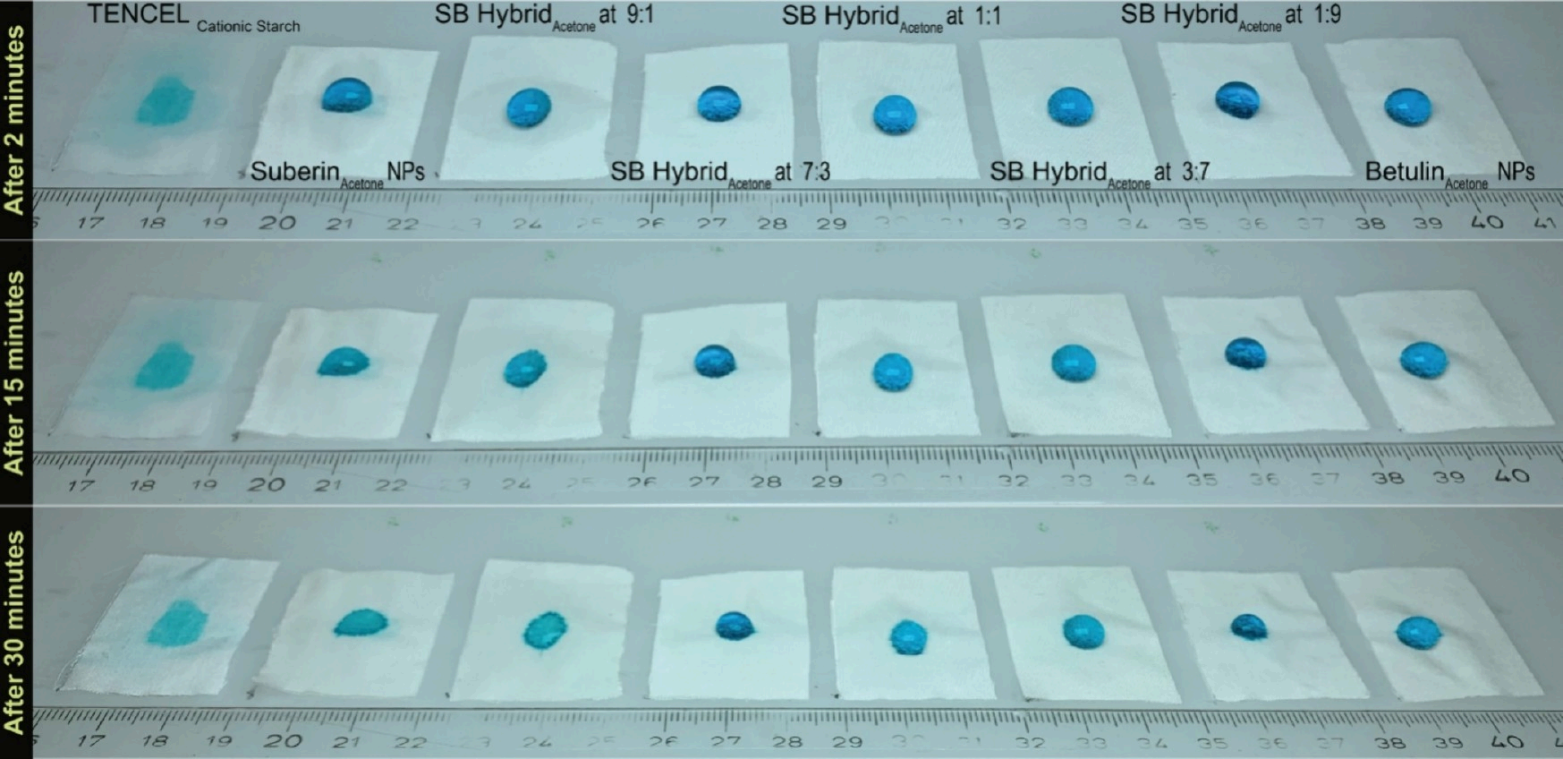
Absorption of water droplets as a function of time on the TENCEL fabric treated with SH NPs, BF NPs, and hybrid NPs.

These findings indicate that BF NPs impart the highest water repellence among the tested samples, thereby providing valuable insights into the varying water-resistant properties of the coated surfaces. This combination of extended water repellency from BF NPs and extreme hydrophobicity from BF crystals (WCA > 160°) makes these materials excellent choices for developing eco-friendly, water-resistant coatings on textiles, paper-based packaging, and wood surfaces that are regularly exposed to moisture. What is particularly impressive is that this high level of water repellency can be achieved through a simple, sustainable process using renewable materials offering a green alternative to conventional synthetic coatings.

### 2.8 Antibacterial Efficacy of SH, BF, and Their Self-Assembled Architectures

The antibacterial activities of SH NPs, BF NPs, BF crystals from acetone, and hybrid SB NPs at (1:1) and (3:7) ratios were tested against Gram-negative K12+pcGLS11 and Gram-positive RN4220+pAT19 luminescent indicator strains. All nanoparticles derived from SH and BF exhibited antimicrobial activity against both and as depicted in Figure 9, except for the BF crystals, which demonstrated no activity against . Overall, the NPs exhibited greater efficacy against in comparison to that against . The SH NPs exhibited the highest level of activity, demonstrating over 58% inhibition of , followed by SB Hybrid (3:7) NPs with >55% inhibition and BF NPs, which showed over 51% inhibition.

**Figure 9:**
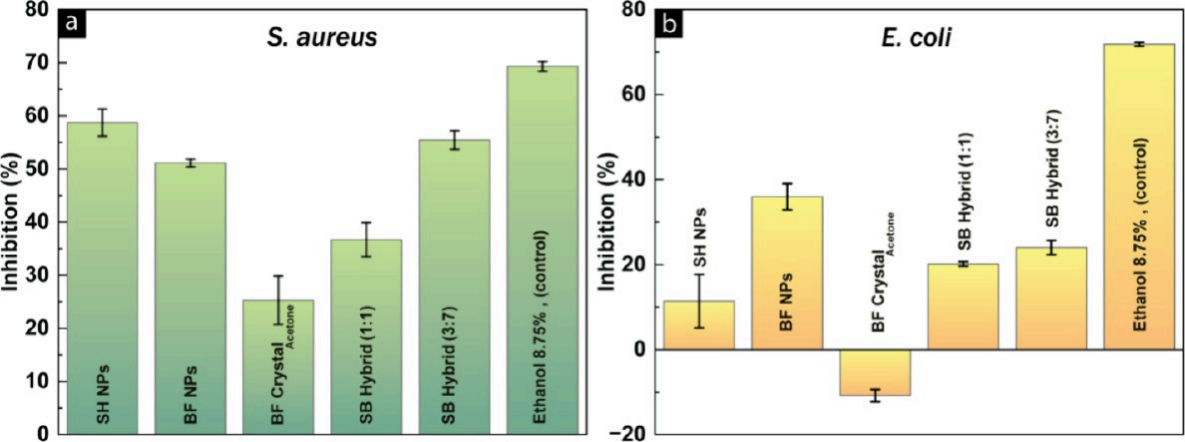
Suspensions of SH NPs, BF NPs, BF crystals from acetone, and hybrid SB NPs (1:1), (3:7) tested with (A) and (B) biosensors and the results are shown for the content of 2.6 mg/mL per sample. Ethanol at 8.75 vol % is shown as a positive reference. Results are presented as averages of inhibition percentages ± standard deviation of three sample replicates. Negative inhibition% indicates that bacteria are able to use the sample as a nutrition.

These results indicate that the antibacterial effectiveness of SH and BF is preserved throughout the extraction process and their transformation into nanoparticles, with statistical analysis confirming highly significant differences in antimicrobial activity between sample types for both (*F*(5,12) = 252.15, *p* < 0.0001) and (*F*(5,12) = 108.81, *p* < 0.0001). Among the tested nanoparticles (NPs) and crystals, BF crystals demonstrated the lowest level of inhibition (>25%) against . Similarly, the SB Hybrid NPs at 1:1 demonstrated lower antibacterial activity against compared to their single constituents, SH NPs and BF NPs. Nevertheless, hybrid NPs at a ratio of 3:1 were able to restore antibacterial efficacy to over 55% inhibition. The differences in antibacterial effectiveness among the NPs and crystals observed can be attributed to two main factors: surface chemistry and particle morphology.^66^ The variances in surface functional groups and their distribution, along with factors such as average particle size, shape, specific surface area, and surface curvature, collectively influence both the antibacterial activity and mechanism.^66^ In the case of BF crystals, the comparatively lower % inhibition of can be attributed to its larger particle size (Figure 4) and the self-assembly process resulting in distinct surface functional groups. On the other hand, the morphological analysis (Figure 5) reveals that SB Hybrid NPs at 1:1 exhibit a needle-shaped morphology, whereas at a 3:7 ratio, the resulting hybrid NPs adopt a spherical morphology similar to that of the individual SH and BF NPs. Therefore, it can be deduced that the variation in NP morphology contributes to the observed differences in antibacterial activity. In the case of , both SH and BF nanoparticles demonstrated a lower percentage of inhibition, with BF NPs showing a maximum antibacterial activity of 36%. The differences in antibacterial efficacy among SH, BF nanoparticles, and crystals against and bacteria could be ascribed to difference in the cell wall structure of the two bacterial strains.^67^ The outer membrane of bacteria potentially functions as a selective barrier, affecting responses to antibacterial agents. These observations align with existing literature, which has shown that suberin exhibits lower activity against in comparison to .^18,23,67–69^ Overall, the bactericidal property of SH and BF nanoparticles against both Gram-positive and negative , along with their tunable size and hydrophobic characteristics, vindicate their potential for developing water-repellent surfaces with inherent antibacterial features.

### 2.9 Thermal Properties of SH, BF, and Their Self-Assembled Architectures

The X-ray diffraction (XRD) analysis provided significant insights into the structural organization and morphology of SH and BF with varying architectures. To establish a comprehensive understanding of the structure–property relationship, thermal analysis utilizing DSC was conducted, alongside XRD observations. The DSC thermograms of initial BF powder, BF crystals obtained from acetone, and BF NPs are presented in Figure 10a. Additionally, Figure 10b displays the thermograms of SH powder, SH NPs, and hybrid SB NPs at various ratios. The DSC thermogram of the SH powder displays a melting endotherm at 66.5 °C, which falls within the reported melting point range for the microcrystalline phase of suberin-derived materials.^16,18^ The broad melting transition of SH can be attributed to its diverse molecular weight distribution, suggesting the presence of crystalline domains and correlating well with the multicomponent nature of the sample.^18^ The melting endotherm of SH NPs shifts toward higher temperature, exhibiting a sharp melting endotherm at 73 °C, accompanied by an increase in the endothermic peak intensity. The elevated endothermic temperature is associated both with the formation of stable NPs and the reduced heterogeneity of the substance.^58^ This shift indicates a well-defined crystalline structure in the NPs compared to that of bulk powder.

**Figure 10:**
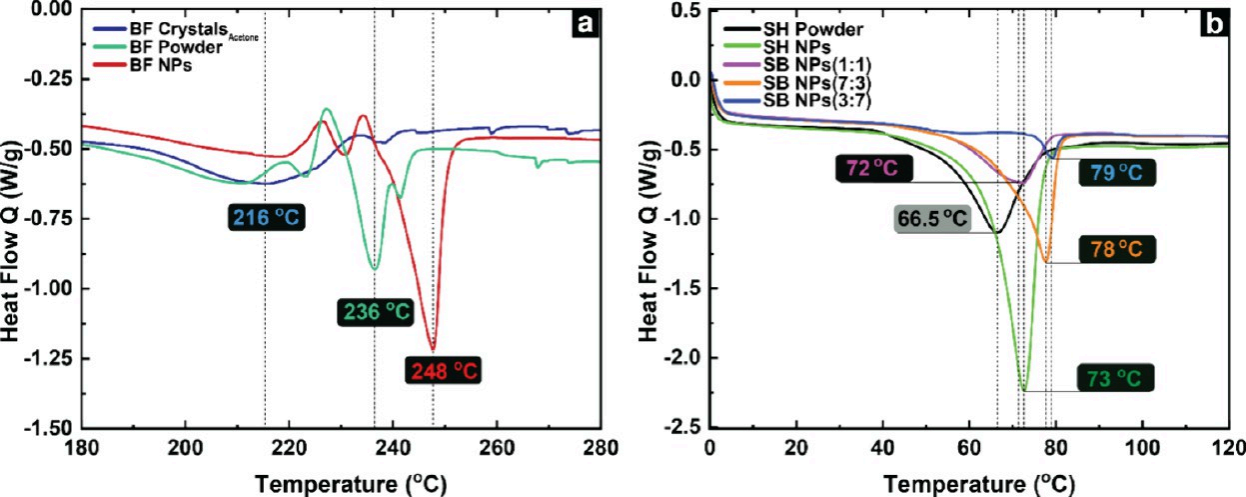
Thermal analysis of (a) various morphologies of BF, (b) SH powder, SH NPs, and hybrid SB NPs using differential scanning calorimetry.

The melting enthalpy (Δ*H*_f_) of SH powder was determined as 68 J/g from the DSC thermogram. A significant increase in Δ*H*_f_ was observed for SH NPs, which reached up to 105.7 J/g. The observed rise in the melting enthalpy can be attributed to the augmented crystal concentration, which is supported by the XRD analysis (Figure 6a).

The DSC thermograms of hybrid SB NPs showed an increasing trend in melting temperature (*T*_m_) with a higher percentage of BF. Hybrid SB NPs prepared at a 3:7 ratio of SH to BF exhibited *T*_m_ at 78 °C, whereas at 7:3 ratio, the *T*_m_ was 79 °C. However, hybrid NPs at 1:1 ratio showed an anomalous *T*_m_ at 72 °C. The increase in melting temperature with higher BF content is attributed to the higher melting point of betulin. As we can observe in Figure 10a, the DSC curve of BF powder displayed an exothermic event at 199 °C attributed to its glass transition temperature (*T*_g_), followed by a crystallization exotherm (*T*_c_) at 218 °C and a melting peak *T*_m_ of 236 °C. The reported betulin melting point ranges from 251 to 262 °C,^70–72^ and the values are influenced by betulin source, fractionation method, and chemical composition. Due to the same reason, the self-assembled BF NPs displayed a *T*_m_ of 248 °C, aligning closely with the literature value 245 °C for orthorhombic betulin,^72^ because of removal of nondissolved residues. These BF NPs also exhibited a *T*_c_ of 226 °C, at higher temperature than the BF powder at 218 °C. On the other hand, the BF crystals obtained from acetone showed a broader melting peak and a lower *T*_m_ of 216 °C, indicative of the polydispersity of crystal domain sizes. This lower melting point and broader peak result from suboptimal crystallization conditions that entrap impurities within the crystal lattice, disrupting molecular arrangement.^72^

The starting BF powder and BF crystals from acetone exhibited identical Δ*H*_f_ values of 24 J/g. Significant increase was observed for the self-assembled BF NPs where Δ*H*_f_ was 36 J/g, in line with the XRD analysis indicating a degree of crystallinity of 88%, following the linear relationship between enthalpy of melting and degree of crystallinity.^73^ This highlights the superior structural refinement achieved through nanoparticle self-assembly. The Δ*H*_f_ values for all of the measured samples are given in Table S5.

## 3 Conclusion

This study provides insights into the supramolecular self-assembly behavior of SH, BF, and their hybrids, outlining key design principles for advancing biobased nanomaterials. A central finding is that chemically heterogeneous and low-purity bark extracts can be transformed into well-defined, stable nanoparticles through solvent-driven assembly, eliminating the need for extensive purification. This enables a more sustainable and economically viable pathway for converting abundant biomass to functional materials. We show that the nanoparticle structure can be tuned by tailoring the solvent environment and leveraging inherent chemical functionalities such as hydroxyl, carboxyl, and epoxy functional groups. These functional moieties govern the noncovalent interactions essential for self-assembly, and their distribution influences nanoparticle stability and architecture. Hybrid assemblies of SH and BF further demonstrate that compositional synergy enables the creation of hierarchical nanostructures.

The resulting nanoparticles exhibit excellent surface functionality, including high hydrophobicity, water repellency, and antimicrobial activity, which is critical for applications in coatings, packaging, and biomedical materials. These functional attributes, achieved without chemical modification, underscore the utility of natural extractives as active components rather than inert additives. Altogether, this work provides a template for designing next-generation biobased self-assembled systems using heterogeneous natural components and mild processing, offering a scalable route toward multifunctional, sustainable nanomaterials. While this work provides insights into the self-assembly behavior of these bark extractives, detailed mechanistic studies using techniques such as MD simulations and advanced spectroscopic methods would be needed to fully understand the self-assembly pathways in these complex mixtures.

## 4 Experimental Section

### 4.1 Chemicals

The solvents, acetone (100%), ethanol (99.5%, Aa), and 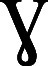-valerolactone (ReagentPlus, 99%) were procured from VWR Chemicals BDH, Anora Industrial, and Sigma-Aldrich, respectively. (Trimethylsilyl chloride (TMCS, >98%) and pyridine (99.8%, anhydrous) were obtained from Merck KGaA. *N*,*O*-bis­(trimethylsilyl) trifluoroacetamide (BSTFA) was obtained from Supelco Analytical.) The Poly-l-lysine (PLL) solution was obtained from Sigma-Aldrich at a concentration of 0.1% (w/v) in H_2_O, with a molecular weight ranging from 150,000 to 300,000 g mol^–1^. Cationic starch (Classic 145) was from Chemigate Oy, Finland. All solvents and chemicals were utilized in their original form upon acquisition.

### 4.2 Extraction of SH and Betulin Extract (BE) Fraction

The SH and betulin extract (BE) fractions were extracted from freshly cut silver birch ( Roth) stems following the method described by Yadav et al., 2024.^74^ The complete details of the method are provided in the Supporting Information.

### 4.3 Supramolecular Self-assembly of SH and BF

Solutions containing 1 wt % of SH and BF in acetone, ethanol, and 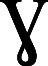-valerolactone were prepared. The mixtures were initially agitated at 600 rpm for a duration of 15 min at 85 °C, followed by continuous stirring at ambient temperature for a period of 12 h to facilitate dissolution. The solutions were ultimately subjected to centrifugation to eliminate any remaining undissolved residues. Centrifugation was performed at 10,000 rpm for 30 min. Self-assembly was achieved by rapidly pouring the dilutions or mixtures into deionized water that was vortex-stirred with a solution-to-water ratio of 1:5 (v/v). Subsequently, the dispersed particles underwent dialysis against water for approximately 48 h using Spectra/Por 1 tubing with a molecular weight cutoff (MWCO) of 6–8 kDa to eliminate the organic solvent. A graphical representation (Scheme S1) of the self-assembly process is provided in the Supporting Information. Dilutions and mixtures in this study were prepared using freshly made solutions following the established protocol to prevent crystallization.

“For hybrid nanoparticle preparation, SH and BF were combined at five different ratios (SH:BF): 9:1, 7:3, 1:1, 3:7, and 1:9. These ratios represent both volume ratios of the solutions and weight ratios of the solutes, as they were prepared by mixing appropriate volumes of solutions at the same concentration (0.2 wt %). For example, for the 1:1 ratio, 1 mL of 0.2 wt % SH in acetone was combined with 1 mL of 0.2 wt % BF in acetone prior to self-assembly. Similarly, other ratios were prepared by adjusting the volumes while maintaining the same total volume. Additionally, a concentration-dependent study was conducted using the 1:1 ratio, with both SH and BF concentrations varied from 0.2 to 1 wt % (0.2, 0.4, 0.6, 0.8, and 1 wt %).”

In order to observe the crystallization of the betulin-rich fraction, solutions were prepared by dissolving BF in acetone and ethanol at concentrations of 1, 2, 3, 4, 5, and 6 wt % in 10 mL of each solvent. The mixture was subjected to overnight stirring at 600 rpm, followed by a stabilization period of 3 weeks to allow for additional crystallization. To prepare the samples for FESEM, the sediment portion of the solution was carefully transferred into 10 mL of deionized (DI) water to facilitate the suspension of the pre-existing crystals. The dispersion was subsequently deposited onto a silica wafer for visualization. The details of thin film preparation are provided in SI.

### 4.4 Particle Size and ζ-Potential Measurement

Particle size and ζ-potential analysis of SH, BF, and the hybrid NPs were measured by using a Zetasizer Nano ZS90 instrument (Malvern Instruments Ltd., UK). A dip cell probe was used, and the Smoluchowski model was employed to calculate the ζ-potential values from the recorded electrophoretic mobility measurements.

### 4.5 Sample Preparation for Water Contact Angle (WCA) Measurements

Prior to analysis, the TENCEL fabric samples measuring 1 cm × 1 cm were excised and subjected to a washing step in ethanol for 5 min, followed by a rinse with deionized water for 5 min to eliminate any potential contaminants present on the fabric. Subsequently, every individual piece of fabric was immersed in a solution of 0.1 wt % cationic starch for 15 min. Following this, the fabric was rinsed for a further 5 min with deionized water. The fabric was immersed in dispersions for 20 min, followed by a subsequent rinsing for 5 min. To ensure adequate coverage, we left the fabric specimens to dry overnight. Subsequently, a second layer of the dispersion was applied, followed by a 5 min rinse with deionized water.

### 4.6 Field Emission Scanning Electron Microscopy (FESEM)

FESEM was conducted using either the Zeiss Sigma VP (Carl Zeiss AG, Oberkochen, Germany) or Tescan Mira3 (Tescan, Brno, Czech Republic) instruments. Triplicate FESEM micrographs were analyzed in FIJI ImageJ to estimate the particle size. One-way ANOVAs (α = 0.05) were performed separately for each solvent system to assess concentration effects. The details are outlined in the Supporting Information.

### 4.7 X-ray Photoelectron Spectroscopy (XPS)

XPS was employed to investigate the variation in the elemental composition among different samples. The measurements were conducted by utilizing a Kratos AXIS Ultra DLD spectrometer (Kratos Analytical, Manchester, UK), which employed an Al Kα monochromatic X-ray source (1486.7 eV). The survey scans were conducted using a step size of 1.0 eV and at an 80 eV analyzer pass energy. On the other hand, high-resolution regional spectra were acquired with a step size of 0.1 eV and at 20 eV pass energy. During the measurement process, the samples were charge-neutralized by using slow electrons emitted from a tungsten filament. The base pressure of the system was recorded to be below 1 × 10–9 Torr. XPS measurements were conducted on each sample, with three repetitions performed on distinct points of the sample surfaces to assess the homogeneity, reliability, and reproducibility of the results. The acquired peaks were adjusted for charge correction in relation to the position of the C 1s component, which represents C–C bonding at 284.8 eV. The XPS data was analyzed using the CasaXPS software.

### 4.8 Differential Scanning Calorimetry (DSC)

Thermal analysis of the SH, BF, and nanoparticle systems was conducted using a TA Instruments DSC 250, which was equipped with an RSC 90 cooling system. The temperature measurements were conducted over a range of 0 to 300 °C, utilizing a heating rate of 10° /min.

### 4.9 X-ray Diffraction (XRD)

To estimate the effect of supramolecular self-assembly onto the crystallinity, X-ray diffraction measurements were conducted using a PANalytical X’PERT PRO MPD Alpha1 instrument with Cu K-alpha1 radiation and a Ge monochromator. The measurement was conducted over a range of 3° to 70° angle (2θ), with a step size of 0.026° and a duration of 300 s per step. During the measurements, the samples underwent a rotation.

### 4.10 Water Contact Angle (WCA)

The wetting characteristics of TENCEL fabric surfaces coated with SH NPs, BF NPs, and crystals were analyzed through the measurement of water contact angle (WCA) using a Theta Flex tensiometer from Biolin Scientific (Gothenburg, Sweden) via the sessile drop method. A droplet of 7 μL of deionized water was placed onto the surface of the coated fabric and imaged for a duration of 1 min, at one image per second rate. Water contact angle data were analyzed using one-way ANOVA followed by Tukey’s HSD posthoc test (α = 0.05).

### 4.11 Water Absorption

In order to assess the performance of TENCEL fabric surfaces treated with SH NPs, BF NPs, and BF crystals, a droplet of DI water (100 μL) was applied onto the treated fabric samples, and visual documentation was obtained through photography. The initial photograph was captured promptly after the droplet was deposited, and subsequent images were taken at specific intervals of 2, 15, 30, 60, and 90 min. The experiment was conducted in a conditioning room, maintained at a temperature of 22 °C and a relative humidity (RH) of 50%. For better contrast, malachite green dye was used to color the DI water.

### 4.12 Gas Chromatography–Mass Spectrometry (GCMS)

The 100 mg portion of the sample was dissolved in 100 mL of 99.5% ethanol. Sonication was employed to enhance the dissolution process. An aliquot of the specimen, which produced an estimated 0.4 mg of dry solids, was subjected to a nitrogen gas stream drying process at a temperature of 50 °C. The dried sample underwent derivatization through the addition of 25 μL of pyridine (Merck KGaA, Darmstadt, Germany), 100 μL of N,O-bis­(trimethylsilyl) trifluoroacetamide (BSTFA, Supelco Analytical, Bellefonte, PA, USA), and 25 μL of trimethylsilyl chloride (TMCS, Merck KGaA, Darmstadt, Germany). The silylation was conducted at a temperature of 70 °C for 45 min. Internal standards, including heneicosanoic acid (C21:0) at a concentration of 0.02 mg/mL, cholesterol at 0.02 mg/mL, cholesteryl heptadecanoate (Ch17) at 0.02 mg mL^–1^, and 1,3-dipalmitoyl-2-oleyl-glycerol (TGstd) at 0.02 mg mL^–1^, were utilized in the study. The silylated samples were analyzed on a GC–MS using an HP6890-5973 GC–MSD instrument (Hewlett-Packard, Palo Alto, CA, USA), with an HP-5 GC column (Agilent Technologies, Inc., Santa Clara, CA, USA; 30 m × 0.25 mm i.d., film thickness 0.25 μm). Helium was used as the carrier gas, and the injection was made in splitless mode. The temperature profile was as follows: 150 °C → 230 °C, 7 °C/min, 230 °C → 310 °C, 4 °C/min, hold time 10 min. The injector temperature was 260 °C and the detector 290 °C. The mass spectra were acquired using electron ionization mode (70 eV), and the fragmentation pattern was subsequently compared to reference standards available in commercial libraries such as NIST14 and Wiley11, as well as those present in our own MS libraries. The sample that underwent silylation was subjected to GC-FID (Shimadzu GC-2010, Kyoto, Japan) with an HP-1 column (Agilent Technologies, Inc., Santa Clara, CA, USA; 15 m × 0.53 mm, 0.15 μm) analysis to determine its group composition. The temperature profile was as follows: 100 °C, hold time 1.5 min, 100 °C → 325 °C, 12 °C/min, hold time 6 min. The temperature profile of the injector was 50 °C, hold time 0.5 min, 50 °C → 340 °C, 200 °C/min, hold time 18 min. The detector temperature was 325 °C.

### 4.13 Nuclear Magnetic Resonance (NMR) Spectroscopy

The ^1^H and HSQC NMR spectra were obtained using a Bruker Avance III 400 MHz spectrometer to record liquid-state NMR spectra. The suberin-rich SH fraction was dissolved in DMSO-*d*_6_, and the betulin-rich BH fraction was dissolved in acetone-*d*_6_. Solvent signals were used for referencing the spectra. Quantitative ^31^P NMR was performed using the same equipment, and samples were prepared according to Granata and Argyropoulos.^75^ Experimental details are described in SI.

### 4.14 Antibacterial Efficacy (Microplate Method)

Tests were conducted according to the procedure described by Tienaho et al.^76^ Data were analyzed using one-way ANOVA for each bacterial species (*n* = 3, α = 0.05). Details are given in SI.

## Supplementary Material



## Notes

Self-assembly process, followed by detailed methodological descriptions covering the extraction of SH and betulin extract (BE) fraction, thin film preparation, field emission scanning electron microscopy (FESEM), nuclear magnetic resonance (NMR) spectroscopy, and antibacterial efficacy using the microplate method; comprehensive data on chemical composition of SH and BF analyzed by GC-FID and GC–MS methods, pH values of aqueous suspensions in different solvent systems, characteristics of BF crystals, atomic percentages of chemical elements, and thermal properties including melting points and enthalpies; and FESEM micrographs showing the morphology of SH and BF nanoparticles at varying concentrations (0.2–1 wt %) in acetone, ethanol, and γ-valerolactone solvent systems (PDF)
