# Supplementary material for: Supramolecular Architectures Based on the Self-Assembly of Suberin Hydrolysate, Betulin, and Their Hybrids
Source: Langmuir. 2025 Jul 15;41(29):19156–72. doi: 10.1021/acs.langmuir.5c01278 (PMC12312147; doi:10.1021/acs.langmuir.5c01278)
Supplement: Supplementary file 1 [file la5c01278_si_001.pdf]

Supramolecular architectures based on the self-assembly of Suberin hydrolysate, Betulin fraction and their hybrids

*Muhammad Farooq, Charlotte Zborowski, Paula A. Nousiainen, Jenni Tienaho, Risto Korpinen, Monika Österberg**

Muhammad Farooq, Charlotte Zborowski, Paula A. Nousiainen, Monika Österberg
Department of Bioproducts and Biosystems, School of Chemical Engineering,
Aalto University, Vuorimiehentie 1, 02150 Espoo, Finland
Jenni Tienaho, Risto Korpinen
Production Systems, Natural Resources Institute Finland (Luke), Viikinkaari 9, FI-00790 Helsinki, Finland
E-mail: muhammad.farooq@aalto.fi

Keywords: self-assembly, suberin hydrolysate, betulin, nanoparticles, hydrophobic

TABLE OF CONTENT

1. **Scheme S1.** A graphical illustration of the self-assembly process
2. Detailed description of methods

2.1 Extraction of Suberin hydrolysate (SH) and betulin extract (BE) fraction
2.2 Thin film preparation
2.3 Field emission scanning electron microscopy (FESEM)
2.4 Nuclear magnetic resonance (NMR) spectroscopy
2.5 Antibacterial efficacy (microplate method)

Table S1. Chemical composition (mg/g) of Suberin Hydrolysate (SH) and Betulin Fraction (BF) analyzed by GC-FID and GC-MS methods after silylation of the mixtures.

Table S2. pH values of the aqueous suspensions of SH and BF NPs obtained at different concentrations from three solvent systems, acetone, ethanol and γ-valerolactone.

Figure S1. FESEM micrographs of Suberin Hydrolysate NPs from acetone, ethanol and γ-valerolactone at 0.2, 0.4, 0.6, 0.8 and 1 wt%.

Figure S2. FESEM micrographs of BF NPs from acetone and ethanol at 0.2, 0.4, 0.6, 0.8 and 1 wt%.

Table S3. Characteristics of Betulin fraction crystals formed from acetone at Different Concentrations

Figure S3. XPS survey scan and C 1S spectra of SH, BF and their different morphologies

Table S4. Summary of the atomic percentages (At %) for the chemical elements found in SH and BF powders and various morphologies

Table S5. Melting point (peak maximum), onset temperature and melting enthalpy of different morphologies of SH and BF powders and various morphologies

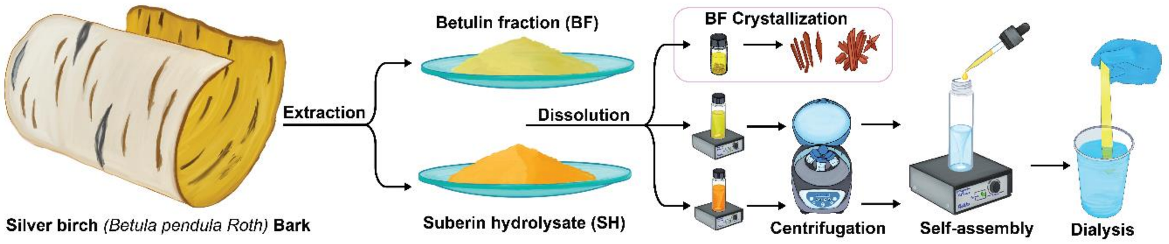

Scheme S1. Steps involved during the supramolecular self-assembly of SH and BF

2. Detailed description of methods

2.1 Extraction of Suberin hydrolysate (SH) and betulin extract (BE) fraction:

Freshly cut silver birch (*Betula pendula* Roth.) stems with a diameter of 200-300 mm from trees grown in Punkaharju, Finland, were harvested and subjected to manual removal of the outer bark. The bark was air-dried at room temperature. The particle size of the separated outer bark was reduced through utilization of a Fritsch Pulverisette cutting mill (Fritsch GmbH, Germany), equipped with a sieve cassette featuring 4 × 4 mm^2 openings. The ground outer bark sample was further subjected to freeze-drying and subsequently stored in dark at room temperature. The ground outer bark was subjected to a two-stage extraction process, first an ethanol extraction, followed by an alkaline hydrolysis in aqueous ethanol.

The oven-dried dry bark (150 g) was introduced into a stirred 2.0 L autoclave (Büchi AG, Uster, Switzerland). A mixture of ethanol and water (1500 mL, 9:1 v/v) was added to the reactor, resulting in a solvent to bark ratio of 10:1. The reactor temperature was elevated from ambient to 90 °C within 15 minutes, followed by extraction process of 30 minutes at 90 °C, with a 150 rpm stirring speed. Next the extraction solution was discharged from the reactor through a brazed plate cooling unit. The collected ethanol/water extract was subjected to vacuum evaporation using a rotary evaporator (Heidolph Instruments GmbH & C. KG, Schwabach, Germany). The residue was subsequently dried in a Heraeus vacutherm vacuum oven (Thermo

Scientific, Thermo Electron LED GmbH, Langenselbold, Germany) at 40 °C for 20 minutes and weighed. The betulin-rich residue (BE1) accounted for approximately 21% of the original dry bark.

The ethanol-extracted bark was further subjected to alkaline hydrolysis in autoclave using the methods described by Korpinen et al., [1] and Kumar et al.[2] with certain modifications. An ethanol/water solution (9:1 v/v) containing 20% NaOH was added based on the original loaded bark dry weight with ratio of used solvent to bark 10:1. The experimental conditions involved elevated temperature at 90°C by maintaining the hydrolysis time of 60 minutes while stirring at a speed of 150 rpm. After the hydrolysis stage the hydrolysate was drained through the cooling unit. The remaining bark residue was washed using an ethanol/water solution (9:1 v/v) at 80°C for 15 minutes. The alkaline hydrolysate and the ethanol/water washing solution were consolidated, and the solvents were removed through vacuum rotary evaporation. Finally, boiling ultrapure water was added to the evaporated residue. This resulted in precipitation of the water-insoluble second betulin fraction, that was filtrated, washed and freeze-dried. The obtained second betulin-rich fraction (BE2) constituted approximately 15% of the original outer bark. The combined betulin-rich-fractions (BF) gave 36% of the original outer bark.

The alkaline filtrate containing suberin fatty acids in their soluble salt form, was then acidified using 2 M sulfuric acid to adjust pH to 4, resulting in the precipitation of the fatty acids. The resulting precipitate was filtered, washed with ultrapure water and freeze-dried. The yield of the suberin fatty acid-enriched fraction (SH) was approximately 26%, as determined from the original outer bark. The designated BH fraction and the SH fraction were utilized in the supramolecular self-assembly experiments and subsequently characterized.

2.2 Thin film preparation:

Thin films were deposited on silica substrates and examined using FESEM to observe self-assembled structures according to the method described by Farooq et al.,[3] with slight modification. The silica substrates underwent a cleaning process that involved immersion in a 1M NaOH solution for 15 seconds, followed by rinsing with deionized water and drying with nitrogen gas. Prior to the deposition process, the substrates underwent a UV/ozone treatment for a duration of 15 minutes. The adsorption method involved depositing a drop of PLL onto the substrate surface as an anchoring polymer, which was left for 30 minutes. The substrate was then rinsed with DI water and dried with nitrogen. A droplet of the dispersion or solution of interest was deposited and left for 30 minutes before being rinsed and dried.

2.3 Field emission scanning electron microscopy (FESEM):

In order to analyze the distinct morphologies of SH and BF architectures, thin films were deposited onto silica wafers and subsequently imaged while affixed to carbon tape. The specimens underwent sputter-coating with a gold-palladium alloy utilizing the Agar high-resolution sputter coater, resulting in the deposition of a 3-5 nm layer. The micrographs were obtained utilizing a secondary electron detector and a working distance of 3-5 mm, with an accelerating voltage of either 3 or 1.5 kV. The Fiji ImageJ software, developed by the Research Services Branch at the National Institutes of Health in Bethesda, Maryland, USA, was utilized for the image analysis.

2.4 Nuclear magnetic resonance (NMR) spectroscopy:

1H were measured using a standard pulse sequence zg30 with 8 scans, spectral width (SW) of 15 ppm, a pulse angle of 30°, an acquisition time (at) of 4 seconds and a pulse delay (d1) of 1 second. HSQC was measured using hsqcetgpsisp.2 pulse sequence with d1 of 2s, and SW of 11 ppm at F2 and 225 ppm at F1 dimension. The spectra were processed with Bruker TopSpin 4.1.4 version software using standard processing parameters. Quantitative 31P NMR were run using a Bruker Avance III 400 MHz spectrometer to determine the hydroxyl groups of both SH and BF samples. The samples were prepared according to Granata and Argyropoulos (1995)[4] using N-Hydroxy-5-norbornene-2,3-dicarboxylic acid imine (10 µmol, 99%, Sigma-Aldrich) in chloroform-d6 as an internal standard. The Bruker inverse-gated decoupling pulse sequence zgig was used to record 128 scans with a 90° pulse angle, 1 s acquisition time, and a relaxation delay d1 of 5 s.

2.5 Antibacterial efficacy (microplate method):

In brief, the constitutively luminescent light signal emitting bacterial biosensor strains *E. coli* K12+pcGLS11 and *S. aureus* RN4220+pAT19 were stored at -80 °C and cultivated for approximately 16 h at 30 °C (*E. coli*) and 37 °C (*S. aureus*) on lysogeny agar plates (LA) (tryptone 10 g/L; yeast extract 5 g/L; NaCl 10 g/L; and agar 15 g/L). The *E. coli* plates were supplemented with 10% (v/v) sterile filtered phosphate buffer (1 M, pH 7.0) and 100 µg/mL of ampicillin and *S. aureus* plates with 5 µg/mL erythromycin. Bacterial stocks were prepared by inoculating a single colony in lysogeny broth with the same supplementations as LA plates. Stocks were again cultivated for approximately 16 h at 300 rpm shaking at 30 °C (*E. coli*) and 37 °C (*S. aureus*). The SH and BF dispersion at 0.2 wt% were diluted in double-distilled water to achieve 50, 25, 2.5, and 6.25 volume % per microplate well. Controls were ethanol in

contents of 8.75 and 17.5 volume % per microplate well (positive control) and double-distilled water (negative control). Samples and controls were pipetted in triplicates of 50 µL into opaque white polystyrene microplates and constant volume of 50 µL bacterial inoculations were added to all microplate wells. The luminescence was then measured using a Varioskan Flash Multilabel device (Thermo Scientific) once every 5 min for 95 min at room temperature, and the plate was briefly shaken before measurements. The results are expressed inhibition percentages (inhibition%) drawn at a time point of 50 min of measurement [5] .Error bars represent the standard deviations between the sample triplicates.

Table S1. Chemical composition (mg/g) of Suberin Hydrolysate (SH) and Betulin Fraction (BF) analyzed by GC-FID and GC-MS methods after silylation of the mixtures.

Number	Compound	Amount in SH (mg/g)	Amount in BF (mg/g)
**1**	Glycerol	0.1	
**2**	Ferulic acid ethyl ester	0.0	
**3**	Palmitic acid (16:0)	0.9	
**4**	Ferulic acid	2.7	
**5**	Linoleic acid (18:2; 9,12)	0.5	
**6**	Oleic acid (18:1; 9)	0.4	
**7**	Stearic acid (18:0)	0.6	
**8**	16-Hydroxy-palmitic acid (16:0)	1.4	
**9**	Arachidic acid (20:0)	0.4	
**10**	Hexadecane-1,16-dioic acid (16:0)	3.3	
**11**	**18-Hydroxy-oleic acid (18:1; 9)**	**47.0**	
**12**	9,10 -Dihydroxy-palmitic acid (16:0)	15.0	
**13**	18-Hydroxy-stearic acid (18:0)	1.1	
**14**	Octadec-9-ene-1,18-dioic acid (18:1; 9)	18.7	
**15**	Octadecane-1,18-dioic acid (18:0)	4.8	
**16**	9,18-Dihydroxy-octadec-9-ene-1,18-dioic acid	6.9	
**17**	Unidentified suberinic acid	17.8	5.3
**18**	**18-hydroxy-9R,10S-epoxy stearic acid (18:0)**	**159.6**	
**19**	20-Hydroxy-eicosenoic acid (20:1; 9)	6.4	
**20**	9,10-Dihydroxystearic acid (18:0)	3.5	
**21**	20-Hydroxy-arachidic acid (20:0)	11.4	
**22**	Eicosene-1,20-dioic acid (20:1;9)	9.9	
**23**	Lignoceric acid (24:0)	0.3	
**24**	**9,10,18-Trihydroxy-stearic acid (18:0)**	**73.8**	
**25**	**22-Hydroxy-docosanoic acid (22:0)**	**51.0**	
**26**	Docosan-1,22-dioic acid (22:0)	11.7	
**27**	24-Hydroxy-lignoceric acid (24:0)	1.3	
**28**	Sitosterol	0.3	5,6
**29**	Sitostanol	0.4	
**30**	Lupeol	14.0	51.7
**31**	Lupenone		5.0
**32**	Lupane-3,20,28-triol		65.3
**33**	Betulonic acid	5.2	12.8
**34**	Erythrodiol	1.8	26.1

**35**	Betulone	2,3	
**36**	**Betulinol**	**175.6**	**623.9**
**37**	Betulinic acid	9.1	39.8
**38**	Unidentified triterpenoid		12.8
**39**	Betulinic aldehyde	10.7	
**40**	Tripeneoid	8.4	
**41**	β-amyrin		1.9
**42**	Monogynol A		72.8
**43**	Oleanolic acid, acetate		11.4
	Total identified	678.3	934.5
	Total unidentified	92.1	58.4
	Total eluted	770.5	980.1

Table S2. pH values of the aqueous suspensions of SH and BF NPs obtained at different concentrations from three solvent systems, acetone, ethanol and γ-valerolactone.

Solvent	Concentration (wt%)	pH	
		SH NPs	BF NPs
Acetone	0.2	4.2	5.9
	0.4	4.3	5.8
	0.6	4.8	5.8
	0.8	4.4	5.6
	1	4.7	5.5
Ethanol	0.2	5.2	6.4
	0.4	4.7	6.3
	0.6	4.3	6.4
	0.8	4.3	6.5
	1	4.8	6.7
γ-Valerolactone	0.2	4.4	0.2
	0.4	4.4	na
	0.6	4.6	na
	0.8	4.4	na
	1	4.4	na

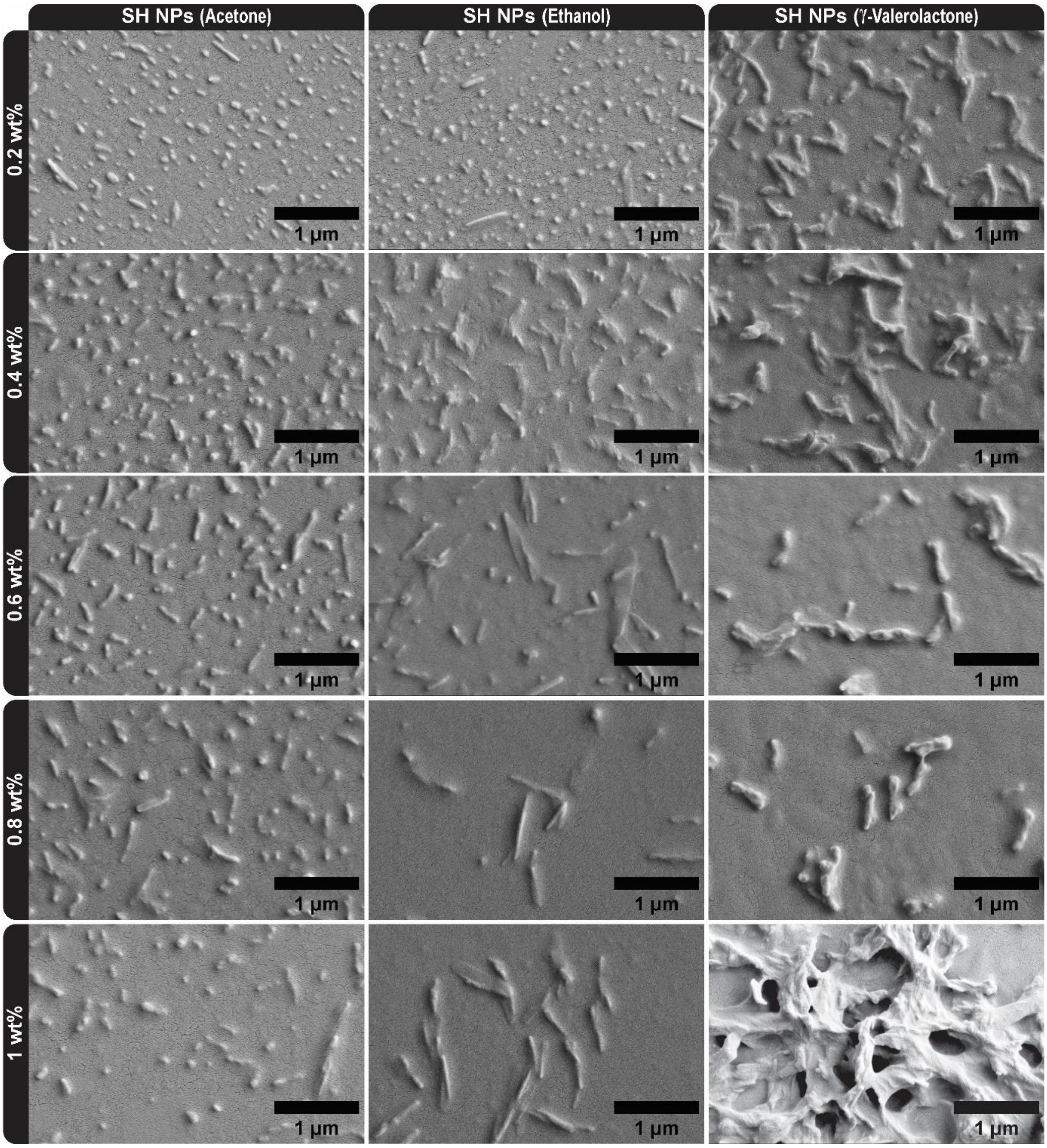

Figure S1. FESEM micrographs of Suberin Hydrolysate NPs from acetone, ethanol and γ-valerolactone at 0.2, 0.4, 0.6, 0.8 and 1 wt%.

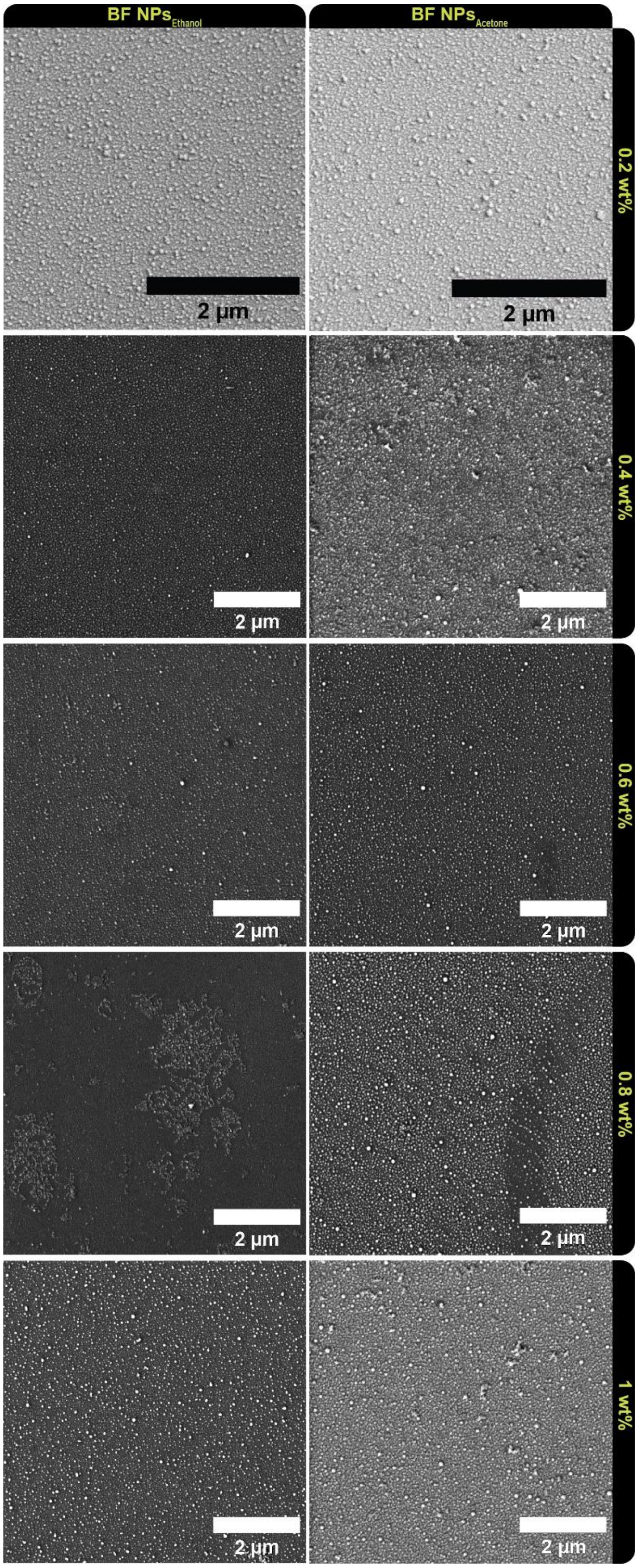

Figure S2. FESEM micrographs of BF NPs from acetone and ethanol at 0.2, 0.4, 0.6, 0.8 and 1 wt%.

Table S3. Characteristics of Betulin fraction crystals formed from acetone at Different Concentrations

Concentration (wt%)	Crystal Length (μm)	Crystal Thickness (nm)
2	1 ± 0.5	60 ± 24
3	0.57 ± 0.27	49 ± 13
4	0.7 ± 0.3	57 ± 21
5	0.86 ± 0.25	66 ± 18
6	5.9 ± 1.5	910 ± 360

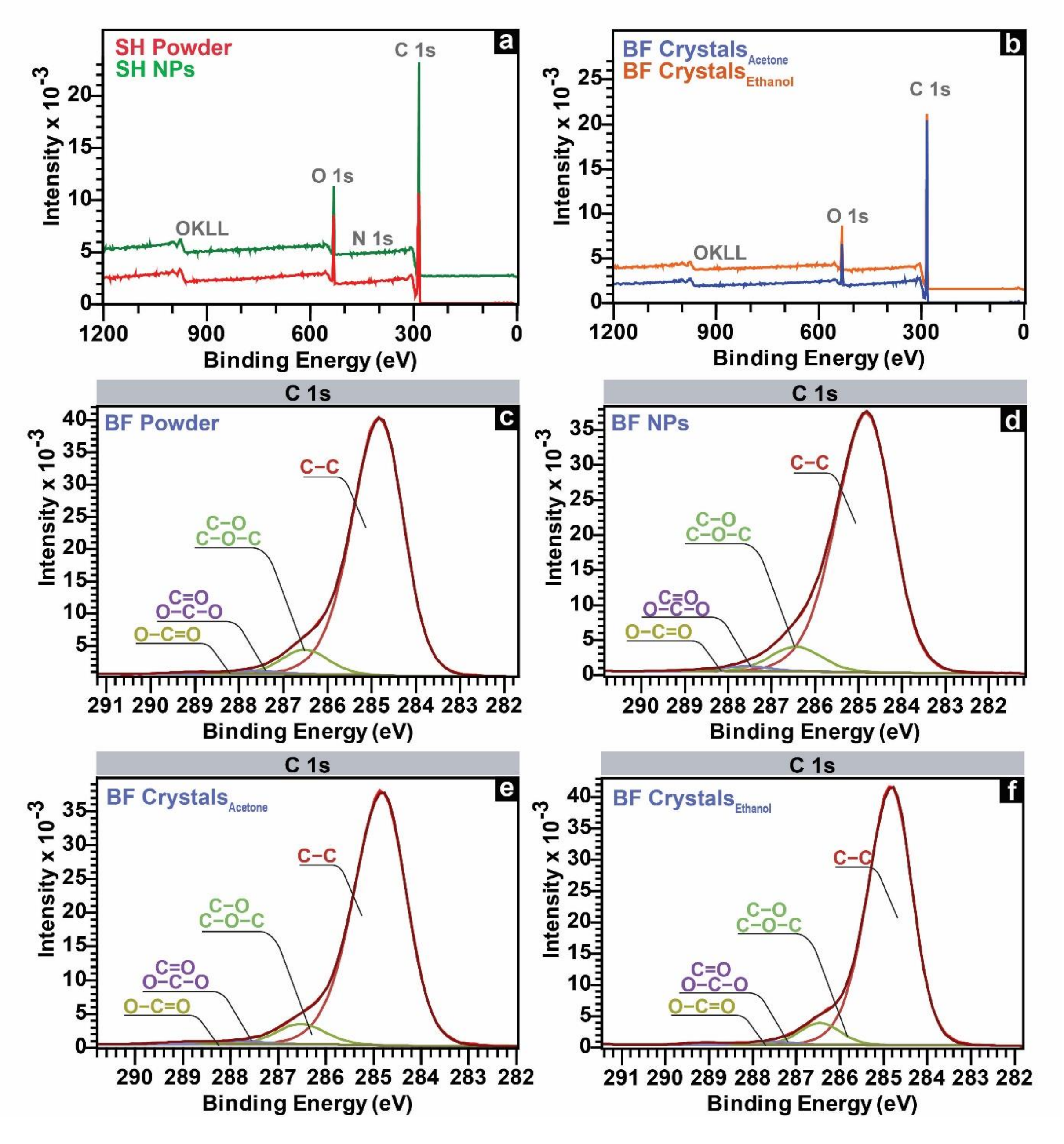

Figure S3. XPS survey scan and C 1S spectra of SH, BF and their different morphologies

Table S4. Summary of the atomic percentages (At %) for the chemical elements found in SH and BF powders and various morphologies

C 1s Components	At.%	SD	At.%	SD	At.%	SD	At.%	SD	At.%	SD	At.%	SD
	C-C		C-O, C-O-C		C=O, O-C-O		O-C=O		π-π*		C-N	
SH powder	73.3	0.4	11.0	0.9	2.6	0.5	3.4	0.1	0.6	0.3	9.2	0.5
SH NPs	86.9	0.2	7.3	0.3	0.7	0.0	2.9	0.0			2.4	0.1
BF powder	89.2	2.2	8.8	1.5	1.4	0.6	0.6	0.1				
BF NPs	90.5	0.4	7.7	0.2	1.5	0.2	0.4	0.0				
BF Crystals $_{Acetone}$	91.2	0.2	6.7	0.2	1.2	0.2	0.8	0.0				
BF Crystals $_{Ethanol}$	91.3	0.0	6.8	0.1	1.1	0.0	0.8	0.0				
SB Hybrid NPs (3:7)	87.4	0.8	9.1	0.6	1.2	0.3	2.3	0.2				

Table S5. Melting point (peak maximum), onset temperature and melting enthalpy of different morphologies of SH and BF powders and various morphologies

Various morphologies	Peak maximum [ºC]	T_{onse} [ºC]	Melting enthalpy [J/g]
SH Powder	66.4	53.4	68
SH NPs	72.6	64.1	105.6
SB NPs (3:7)	79	75.6	4.2
	236	226	41.2
SB NPs (1:1)	72	55	35.6
	229.4	222.1	21.8
SB NPs (7:3)	77.6	68.7	60.3
	227.3	218.1	4.54
BF Powder	236.4	230	23.9
BF Crystals$_{Acetone}$	216.3	193.3	23.6
BF NPs 1	247.7	231	35.6

References

1. Korpinen, R. I. *et al.* The hydrophobicity of lignocellulosic fiber network can be enhanced with suberin fatty acids. *Molecules* 24, 1–14 (2019).
2. Kumar, A., Korpinen, R., Möttönen, V. & Verkasalo, E. Suberin Fatty Acid Hydrolysates from Outer Birch Bark for Hydrophobic Coating on Aspen Wood Surface. *Polymers (Basel)* 14, 1–14 (2022).
3. Farooq, M. *et al.* Well-Defined Lignin Model Films from Colloidal Lignin Particles. *Langmuir* 36, 15592–15602 (2020).
4. Granata, A. & Argyropoulos, D. S. 2-Chloro-4,4,5,5-tetramethyl-1,3,2-dioxaphospholane, a Reagent for the Accurate Determination of the Uncondensed and Condensed Phenolic Moieties in Lignins. *J Agric Food Chem* 43, 1538–1544 (1995).
5. Tienaho, J. *et al.* Salix spp. Bark Hot Water Extracts Show Antiviral, Antibacterial, and Antioxidant Activities—The Bioactive Properties of 16 Clones. *Front Bioeng Biotechnol* 9, (2021).
